# USP10/XAB2/ANXA2 axis promotes DNA damage repair to enhance chemoresistance to oxaliplatin in colorectal cancer

**DOI:** 10.1186/s13046-025-03357-z

**Published:** 2025-03-11

**Authors:** Xingwu Liu, Shaoming Zhang, Yue An, Boyang Xu, Guanyu Yan, Mingjun Sun

**Affiliations:** 1 Department of Gastroenterology, The First Hospital of China Medical University, Shenyang, Liaoning China; 2 Department of Endoscopy, The First Hospital of China Medical University, Shenyang, Liaoning China

**Keywords:** Colorectal cancer, Oxaliplatin resistance, DNA damage repair, Deubiquitination, XAB2

## Abstract

**Background:**

Oxaliplatin-based chemotherapy is the first-line treatment for colorectal cancer (CRC). However, oxaliplatin resistance remains a major challenge contributing to treatment failure and poor prognosis. An increased capacity for DNA damage repair is a key mechanism underlying oxaliplatin resistance. Although XPA binding protein 2 (XAB2) is implicated in various DNA damage repair mechanisms, its specific role in mediating oxaliplatin resistance remains unclear.

**Methods:**

XAB2 was identified through analysis of public datasets. Western blot analysis and immunohistochemistry were performed to evaluate XAB2 expression, while survival analysis was performed to assess its clinical significance in CRC. Functional experiments were then conducted to assess the impact of XAB2 on proliferation, DNA damage repair, and oxaliplatin resistance in CRC. RNA sequencing (RNA-seq) and Chromatin immunoprecipitation-sequencing (ChIP-seq) were used to identify XAB2 target genes. Co-immunoprecipitation (Co-IP) and mass spectrometry were used to identify the proteins interacting with XAB2. Dual-luciferase reporter assays, ChIP-qPCR, Co-IP, ubiquitination site mass spectrometry, and ubiquitin assays were used to analyse the interactions and potential mechanisms involving XAB2, Annexin A2 (ANXA2), and ubiquitin-specific protease 10 (USP10).

**Results:**

XAB2 was found to be expressed in CRC and was associated with poor prognosis in patients with CRC. XAB2 promoted CRC cell proliferation and enhanced oxaliplatin resistance by promoting DNA damage repair. Mechanistically, CRC cells treated with oxaliplatin exhibited increased USP10 nuclear expression. USP10 bound to XAB2 and deubiquitinated XAB2 K48-linked polyubiquitination at K593, thereby stabilising XAB2 by reducing its degradation via the ubiquitin-proteasome pathway. XAB2 upregulates ANXA2 expression at the transcriptional level by binding to the ANXA2 promoter, thereby promoting DNA damage repair, mitigating oxaliplatin-induced DNA damage, and enhancing oxaliplatin resistance.

**Conclusions:**

In summary, this study demonstrates that the USP10/XAB2/ANXA2 axis promotes proliferation, DNA damage repair, and oxaliplatin resistance in CRC. These findings uncover a novel mechanism of oxaliplatin resistance in CRC and suggest potential therapeutic targets for improving the efficacy of oxaliplatin in CRC treatment.

**Supplementary Information:**

The online version contains supplementary material available at 10.1186/s13046-025-03357-z.

## Background

In 2022, more than 1.92 million new cases of colorectal cancer (CRC) and over 900,000 deaths from CRC were reported worldwide [1]. Most patients with early-stage CRC can be effectively treated through surgery. However, approximately 20% of patients are initially diagnosed with metastatic CRC, and up to 50% of those with localised lesions will eventually progress to metastatic CRC [2]. Therefore, combining chemotherapy is crucial for patients with CRC. The FOLFOX regimen—oxaliplatin combined with 5-fluorouracil and calcium folinate—is the current first-line chemotherapy for CRC, effectively alleviating disease progression in patients with advanced disease. However, a significant limitation of the regimen is acquired resistance, which is a major cause of therapeutic failure [3]. Various biological processes, including cell apoptosis and DNA damage repair, are thought to contribute to oxaliplatin resistance [4, 5]; however, the molecular mechanisms underlying oxaliplatin resistance in CRC remain unclear and require urgent investigation to identify potential biomarkers and therapeutic targets.

XPA binding protein 2 (XAB2), which contains the tetratricopeptide repeat motif, plays a crucial role in pre-mRNA splicing, cellular senescence, and DNA damage repair [6, 7]. In recent years, the link between XAB2 and the DNA damage response (DDR) has garnered increasing attention. XAB2 has been shown to be closely associated with DNA damage repair mechanisms, including homologous recombination and nucleotide excision repair [8, 9]. Additionally, DDR is closely linked to tumor biology. On the one hand, DDR is essential for maintaining genomic stability [10]. In normal cells, DDR defects may lead to gene mutations, increased genomic instability, and, ultimately, cancer [11]. On the other hand, DDR deficiencies in tumor cells provide an opportunity for tumor treatment by increasing sensitivity to DNA-damage-inducing drugs. Given the role of XAB2 in DDR, it may be implicated in cancer development and resistance to DNA-damage-inducing chemotherapy medications.

Ubiquitination occurs within the ubiquitin-proteasome system (UPS) [12]. Ubiquitin-protein ligases and deubiquitinases (DUBs) counteract each other to maintain balance in the ubiquitination process. Among the various types of DUBs, ubiquitin-specific proteases (USPs) constitute the largest proportion [13]. Increasing evidence links USPs to tumor progression. USP10, a key member of the USP family, has been shown to play a role in various biological processes in CRC, including proliferation, metastasis, and stemness [14–16]. However, the specific targets and biological functions of USP10 in CRC require further investigation. Understanding the specific targets and roles of USP10 in CRC could lead to innovative therapeutic opportunities.

Annexin A2 (ANXA2) promotes the progression of several cancers, including oesophageal, pancreatic, and gastric cancers [17–19]. High ANXA2 expression in CRC is associated with poor prognosis in patients [20]. Additionally, ANXA2 promotes proliferation, epithelial-mesenchymal transition, and chemotherapy resistance in CRC [21–23]. Given its multifaceted role, ANXA2 may serve as both a diagnostic and prognostic biomarker as well as a potential therapeutic target for CRC treatment. Therefore, studying the association between ANXA2 and CRC cellular behaviour is crucial for understanding ANXA2 function in CRC and may provide novel scientific insights for improving human health.

In this study, CRC cells treated with oxaliplatin exhibited an increase in nuclear expression of USP10. USP10 bound to XAB2 and deubiquitinated it at K593, thereby stabilising XAB2 by reducing its degradation through the ubiquitin-proteasome pathway. XAB2 upregulated ANXA2 expression at the transcriptional level by binding to its promoter, thereby promoting proliferation and DDR in CRC, facilitating oxaliplatin-induced DNA damage repair, and enhancing oxaliplatin resistance. In summary, this study aimed to provide insights into the role of XAB2 in CRC, revealing the potential of targeting the USP10/XAB2/ANXA2 axis to enhance chemotherapy sensitivity in CRC.

## Methods

### Cell lines and cell culture

HEK-293T cells (JNO-H0488), the normal human colonic epithelial cell line NCM460 (JNO-H0138), and human CRC cell lines HCT116 (JNO-H0125), SW480 (JNO-H0143), SW620 (JNO-H0144), RKO (JNO-H0140), and HT29 (JNO-H0131) were purchased from Guangzhou Jennio Biotech (Guangzhou, China). Oxaliplatin-resistant CRC cell line HCT116/L-OHP (MXC469) was purchased from Shanghai MEIXUAN Biological Technology (Shanghai, China). All cell lines tested negative for mycoplasma contamination. HEK-293T and NCM460 cells were cultured in high-glucose Dulbecco’s modified Eagle’s medium (DMEM), and all other cells were cultured in RPMI 1640 medium. All cells were cultured in 10% foetal bovine serum and 1% penicillin/streptomycin with 5% CO_2_ at 37 °C.

### Human tissues

Fifty pairs of paraffin-embedded CRC tissues and corresponding adjacent non-neoplastic tissues were collected in this study to validate XAB2 expression through immunohistochemistry (IHC). Additionally, eight pairs of fresh CRC tissues and adjacent non-neoplastic tissues were collected. This study received approval from the Research Ethics Committee of the First Affiliated Hospital of China Medical University, and informed consent was obtained from all enrolled patients.

### In vivo xenografts

Approximately 2 × 10^6^ cells were resuspended in 100 µl PBS and subcutaneously injected into 5-week-old female BALB/c nude mice (hfkbio, Beijing, China). The length (L) and width (W) of the tumors were measured weekly, and the tumor size was calculated according to the formula: V = 1/2×L×W^2^. After 4 weeks, the mice were euthanized, and xenograft tumor tissues were collected for subsequent analyses.

### Statistical analysis

Statistical Package for the Social Sciences (SPSS) 21.0 and GraphPad Prism 9.2.0 were used for statistical analyses. All data are presented as mean ± standard deviation (SD). For data following a normal distribution, the Student’s t-test was used to compare two independent groups, while a one-way analysis of variance (ANOVA) was used for the comparison of three or more groups, followed by Sidak multiple comparisons test for univariate comparisons. Pearson’s chi-square test was used to analyse the association between XAB2 expression and clinicopathological parameters. The Kaplan-Meier method and log-rank test were used to assess patient survival rates. Statistical significance was set at a threshold of *P* < 0.05.

A detailed explanation of the methods can be found in Additional file 1.

## Results

### XAB2 is highly expressed in CRC and associated with poor prognosis in patients with CRC

**Fig. 1:**
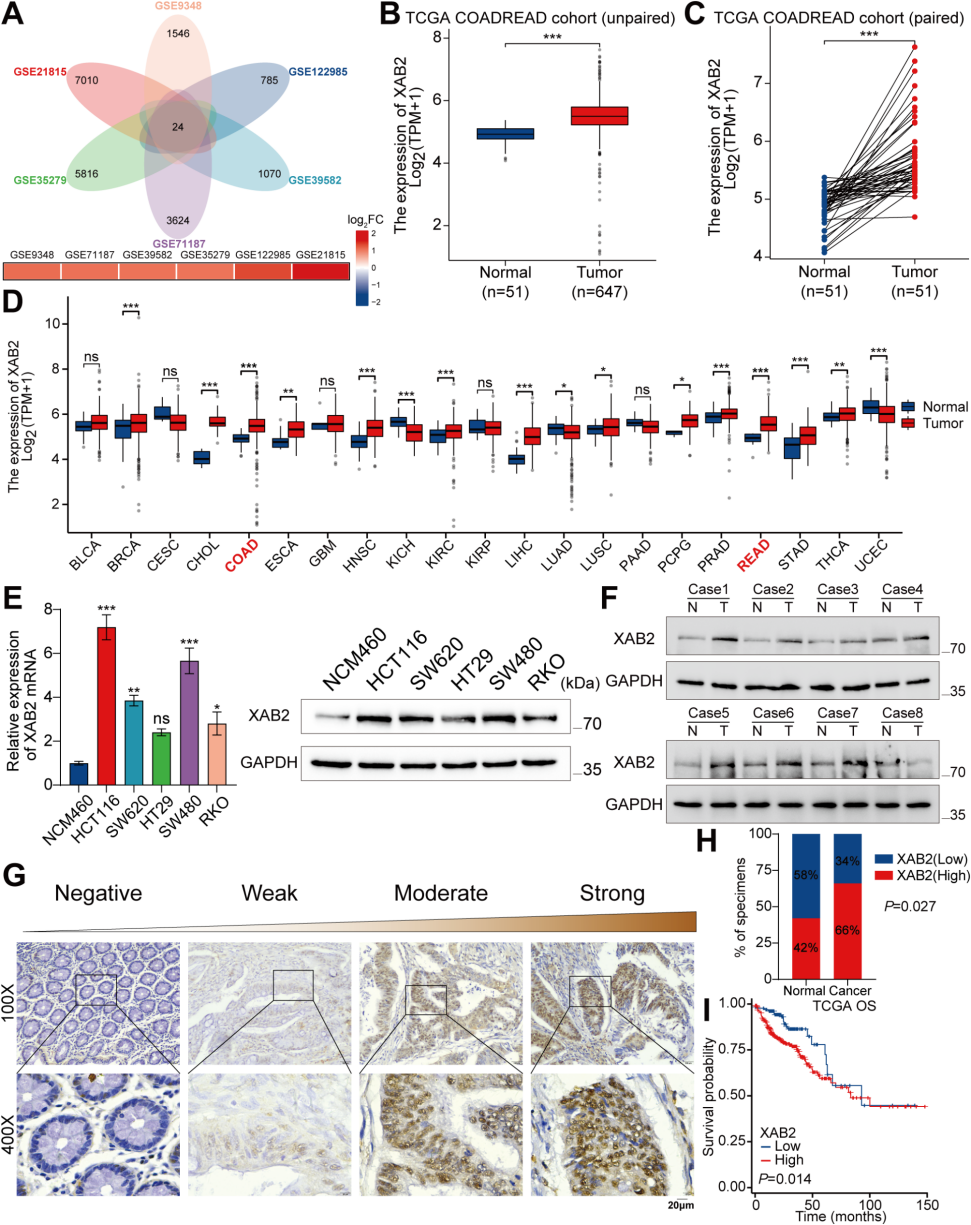
XAB2 is highly expressed in CRC and is associated with poor prognosis in patients with CRC. **A** Overlap of differentially expressed (adjusted *P* value < 0.05 and|logFC|≥1) oncogenes from six datasets and expression levels of XAB2 in six datasets. **B**-**C** Expression levels of XAB2 in unpaired and paired COADREAD cohorts from TCGA. **D** Expression levels of XAB2 in unpaired samples grouped by cancer type from TCGA. **E** qRT-PCR (left) and western blot (right) analysis of XAB2 expression in the normal human colonic epithelial cell line NCM460 and CRC cell lines. **F** Western blot analysis of XAB2 expression in eight pairs of fresh CRC and adjacent non-neoplastic tissues. **G** Representative XAB2 IHC staining images in CRC and adjacent non-neoplastic tissues. **H** Statistical analysis of XAB2 expression in CRC and adjacent non-neoplastic tissues. **I** Kaplan–Meier analysis showing OS curves of patients with CRC stratified by high versus low XAB2 expression from TCGA. Data are presented as mean ± SD (*P < 0.05, **P < 0.01, ***P < 0.001)

Open-access Gene Expression Omnibus datasets (GSE9348, GSE122985, GSE39582, GSE71187, GSE35279, and GSE21815) were analysed to identify potential oncogenes involved in CRC progression [24–29]. The results from these six datasets were intersected, yielding 24 differentially expressed oncogenes, among which XAB2 is the only gene whose expression or biological function has not yet been studied in CRC. The expression level of XAB2 in each dataset was depicted by a heatmap (Fig. 1A). The high expression of XAB2 was subsequently validated using data from unpaired and paired colorectal adenocarcinoma (COADREAD) cohorts in The Cancer Genome Atlas (TCGA) (Fig. 1B-C). Based on the TCGA dataset, XAB2 was found to be highly expressed in various cancer types, including colon adenocarcinoma (COAD) and rectal adenocarcinoma (READ) (Fig. 1D). Quantitative real-time PCR (qRT-PCR) and western blot analyses revealed that XAB2 expression at both mRNA and protein levels were significantly higher in CRC cells than that in normal colonic epithelial cells (Fig. 1E). Furthermore, analysis of eight pairs of fresh CRC tissues and adjacent non-neoplastic tissues, demonstrated higher XAB2 protein levels in CRC tissues than that in adjacent non-neoplastic tissues (Fig. 1F), which was also confirmed by IHC. Representative images of various XAB2 expression levels are shown in Fig. 1G. Notably, high XAB2 protein expression was observed in 66% (33/50) of the CRC tissues compared to only 42% (21/50) in adjacent non-neoplastic tissues (Fig. 1H). Next, the association between XAB2 expression levels and prognosis in patients was analysed. Higher XAB2 levels were significantly correlated with higher T stage (*P* = 0.014) and higher TNM stage (*P* = 0.022) (Table S1). Additionally, analysis using the TCGA and Kaplan-Meier plotter website (http://kmplot.com/analysis/) revealed that higher XAB2 levels were associated with shorter overall survival (OS) and progression-free survival (PFS) (Fig. 1I and Fig. S1A). A nomogram was constructed based on clinical parameters (TNM stage, age, residual tumor, CEA level, and XAB2 expression) to predict 1-, 3-, and 5-year OS in patients with CRC (Fig. S1B). The bias-corrected line of the calibration plot was closely aligned with the ideal (Fig. S1C). The time-dependent receiver operating characteristic (ROC) curve of the nomogram showed that the area under the curve (AUC) for predicting the 1-, 3-, and 5-year OS in patients with CRC was 0.824, 0.846, and 0.774, respectively (Fig. S1D). Collectively, these findings strongly suggest that XAB2 is highly expressed in CRC and is a potential predictive biomarker for OS in CRC.

### XAB2 promotes CRC cell proliferation in vitro and in vivo

**Fig. 2:**
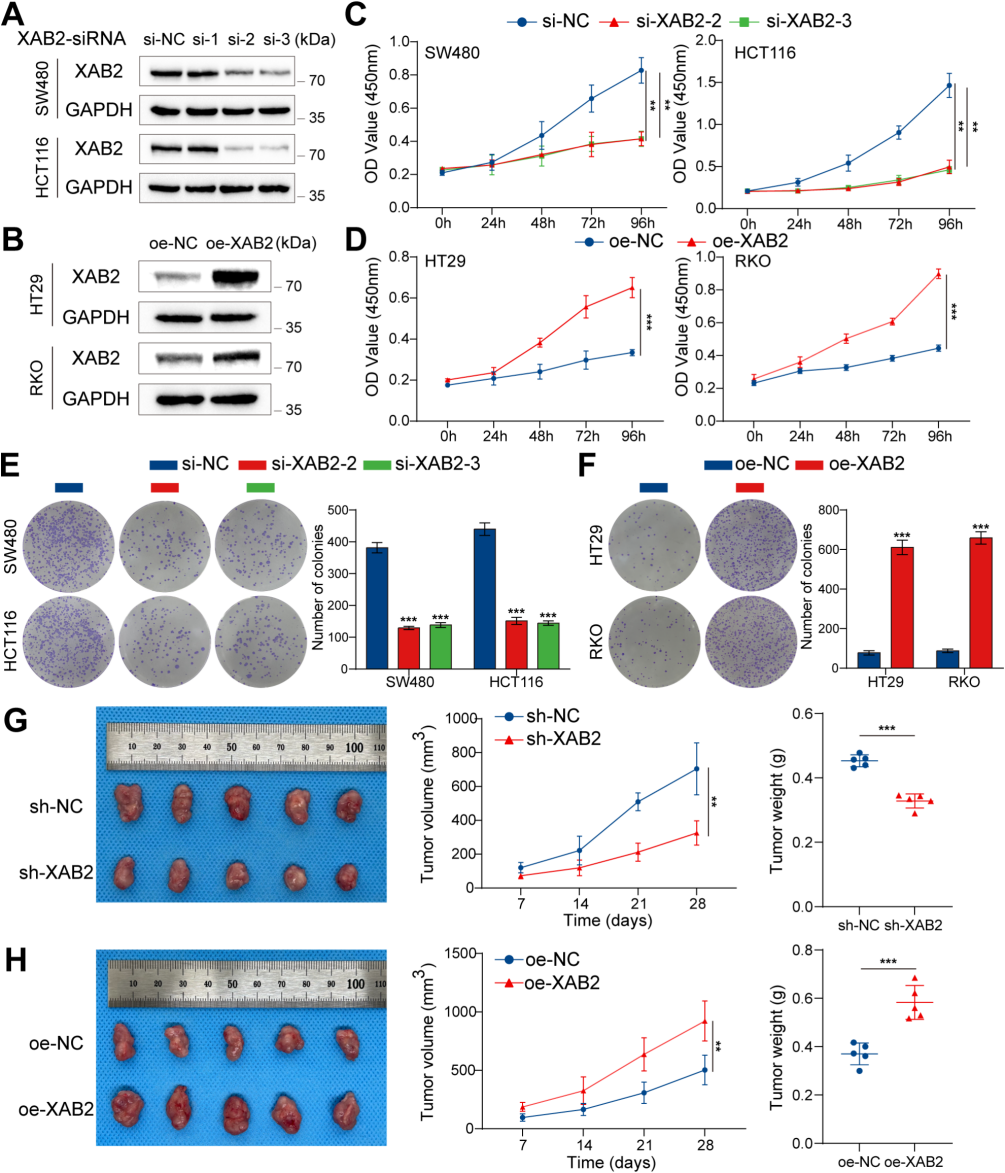
XAB2 promotes CRC cell proliferation in vitro and in vivo. **A** Knockdown of XAB2 in SW480 and HCT116 cells confirmed by western blot analysis. **B** Overexpression of XAB2 in HT29 and RKO cells confirmed by western blot analysis. **C**-**F** Proliferative ability of cells with XAB2 knockdown or overexpression determined by CCK8 assay (**C**-**D**) and colony formation assay (**E**-**F**). **G**-**H** Transplanted xenografts derived from cells with sh-NC and sh-XAB2 were established in BALB/c nude mice (*n* = 5). Tumor volume and weight were measured. Data are presented as mean ± SD (**P < 0.01, ***P < 0.001)

To elucidate the role of XAB2 in CRC, we used siRNAs to knockdown XAB2 in CRC cells (HCT116 and SW480) with higher basal XAB2 expression and overexpressed XAB2 in CRC cells (HT29 and RKO) with lower basal expression. Western blot analysis confirmed the effectiveness of XAB2 knockdown and overexpression (Fig. 2A-B). Cell Counting Kit-8 (CCK8) and colony formation assays revealed that XAB2 knockdown significantly inhibited cell proliferation, while XAB2 overexpression significantly enhanced this effect (Fig. 2C-F). Next, SW480 cells with stable XAB2 knockdown and RKO cells with stable XAB2 overexpression were established and subcutaneously injected into nude mice to evaluate the effect of XAB2 on CRC cell proliferation in vivo. Consistently, XAB2 knockdown significantly inhibited tumor growth, while XAB2 overexpression significantly promoted tumor growth (Fig. 2G-H). Moreover, IHC revealed that the ratio of Ki67-positive cells and PCNA-positive cells was higher in the XAB2 overexpression group, while the ratio was lower in the XAB2 knockdown group, supporting the role of XAB2 in promoting CRC tumor growth (Fig. S2).

### XAB2 increases the resistance of CRC cells to oxaliplatin by promoting DNA damage repair

**Fig. 3:**
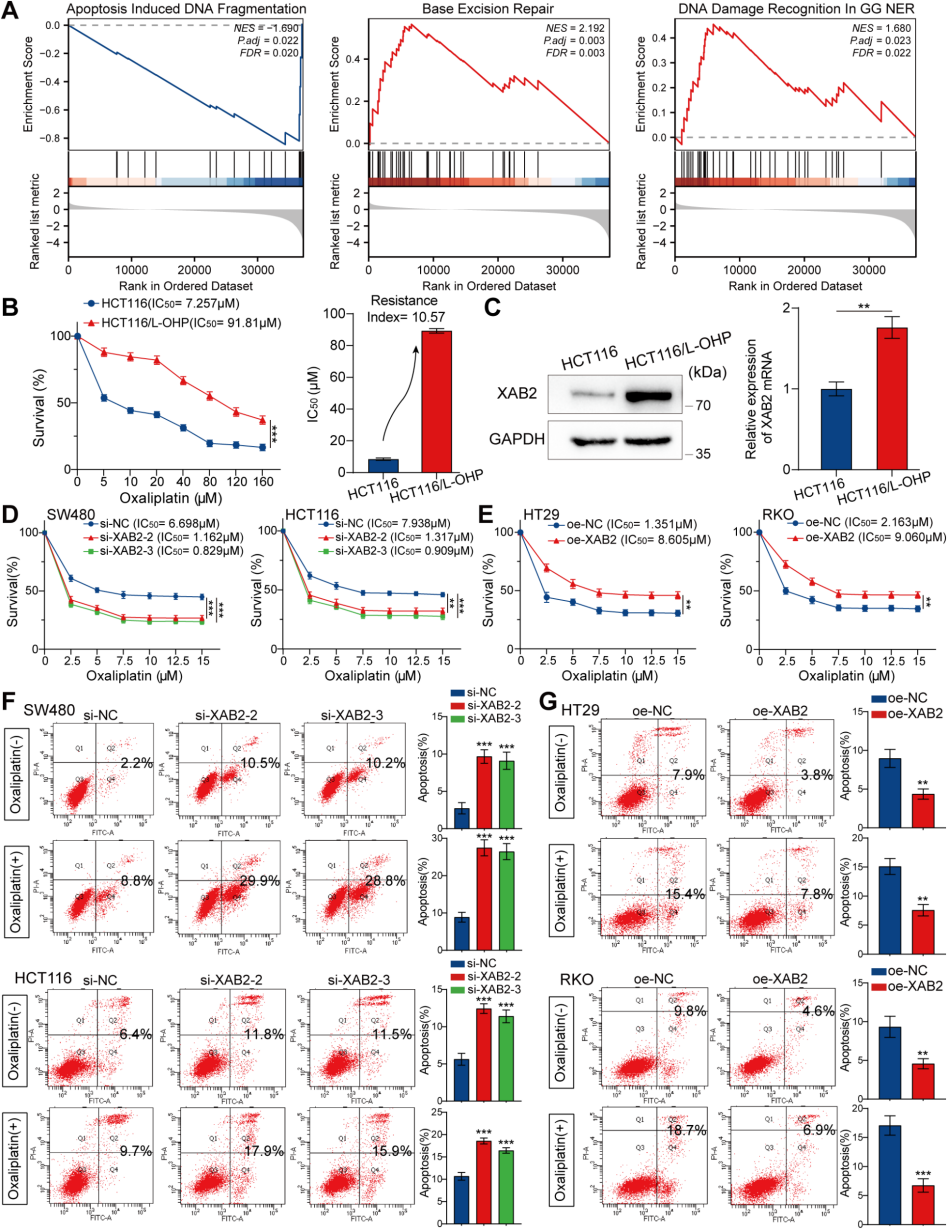
XAB2 increases the resistance of CRC cells to oxaliplatin. **A** GSEA performed using TCGA database, showing XAB2-related enrichment plots. **B** HCT116 and HCT116/L-OHP cells treated with varying concentrations of oxaliplatin for 48 h. Cell viability was analysed using CCK8 assay, and IC50 values were presented. The resistance index was calculated by dividing the IC50 for HCT116/L-OHP cells by that of HCT116 cells. **C** Western blot (left) analysis and qRT-PCR (right) of XAB2 expression in CRC cell line HCT116 and oxaliplatin-resistant CRC cell line HCT116/L-OHP. **D**-**E** CRC cells treated with varying concentrations of oxaliplatin for 48 h. Cell viability was analysed using CCK8 assay, and IC50 values were presented. **F**-**G** Flow cytometry assays assessing the effect of XAB2 on apoptosis of cells treated with or without oxaliplatin (7.5 µM) for 48 h. Data are presented as mean ± SD (**P < 0.01, ***P < 0.001)

Oxaliplatin is a first-line chemotherapy drug for CRC, and DNA damage repair has been implicated in oxaliplatin resistance [30, 31]. Gene set enrichment analysis (GSEA) of the TCGA database indicated a relationship between XAB2 and apoptosis, as well as DNA damage repair (Fig. 3A). Therefore, we hypothesized that XAB2 could influence the response of CRC cells to oxaliplatin. We first performed CCK8 assays to assess oxaliplatin sensitivity in both HCT116 and HCT116/L-OHP cells. The results indicated a significant increase in oxaliplatin resistance in the HCT116/L-OHP cells, as evidenced by a resistance index of 10.57 (Fig. 3B). Next, we detected the expression level of XAB2 in both HCT116 and HCT116/L-OHP cells. The results showed that the expression level of XAB2 in HCT116/L-OHP cells was significantly higher than that in HCT116 cells, suggesting that XAB2 may be related to oxaliplatin resistance (Fig. 3C). In light of the above-obtained results, we next performed CCK8 and flow cytometry assays to evaluate the potential effect of XAB2 expression levels on the response of CRC cells to oxaliplatin. The results showed that XAB2 knockdown increased CRC cell sensitivity to oxaliplatin, as evidenced by lower half-maximal inhibitory concentration (IC50) values and a higher apoptotic rate (Fig. 3D and F). Conversely, XAB2 overexpression rendered CRC cells more resistant to oxaliplatin, with higher IC50 values and lower apoptotic rates (Fig. 3E and G).

**Fig. 4:**
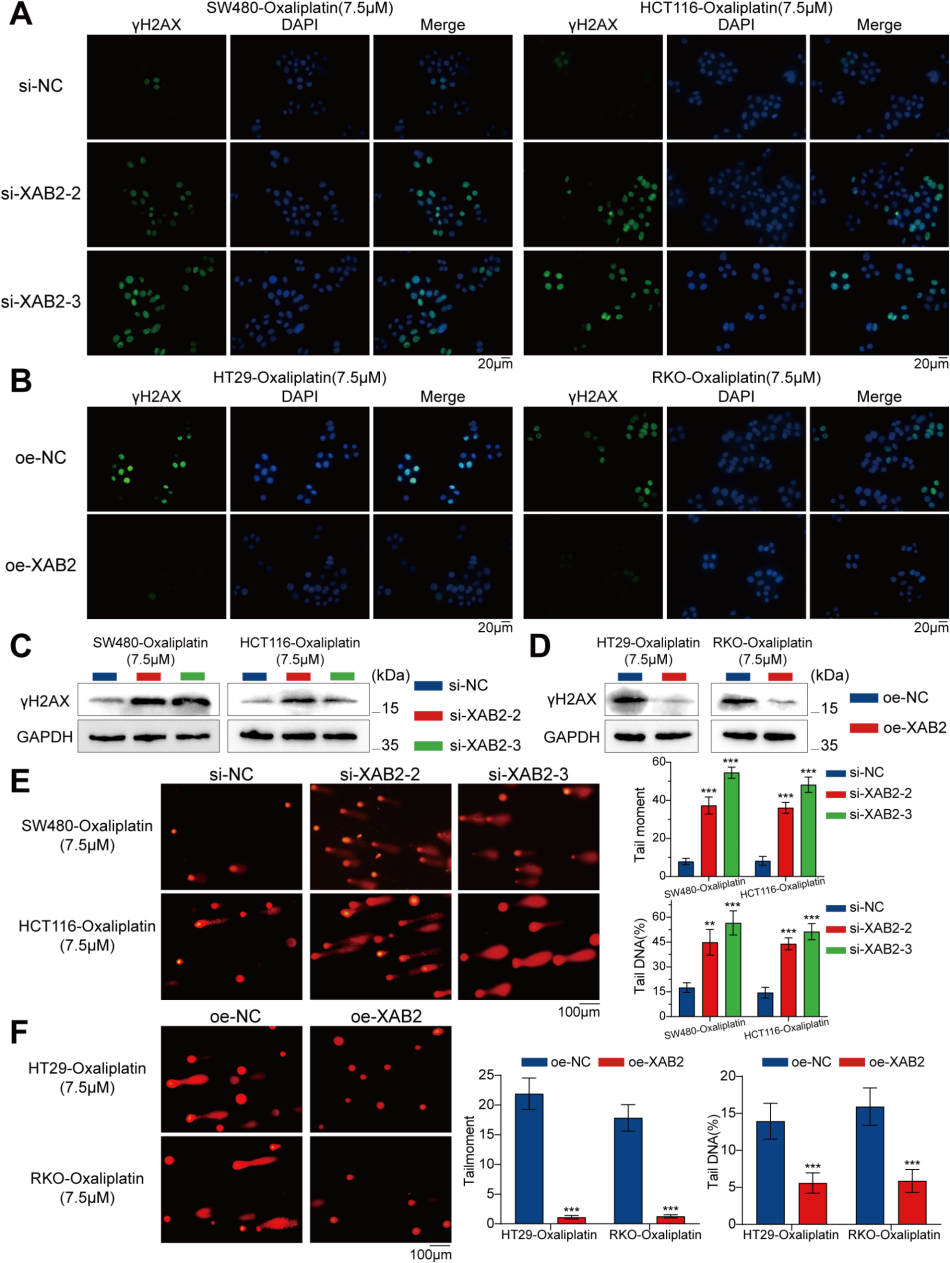
XAB2 enhances DNA damage repair of CRC cells. **A**-**B** Distribution of γH2AX in CRC cells treated with oxaliplatin (7.5 µM) for 24 h analysed via IF. γH2AX is stained green, and the nucleus is stained blue. Scale bar = 20 μm. **C**-**D** Expression levels of γH2AX in CRC cells treated with oxaliplatin (7.5 µM) for 24 h analysed using western blot analysis. **E**-**F** Representative images (left) and bar charts (right) from alkaline comet assays of control cells, XAB2-overexpressing cells, and XAB2 knockdown cells treated with oxaliplatin (7.5 µM) for 48 h. Scale bar = 100 μm. Data are presented as mean ± SD (**P < 0.01, ***P < 0.001)

To investigate the effect of XAB2 on oxaliplatin-induced DNA damage, the level of DNA damage marker - γH2AX was assessed using immunofluorescence (IF) and western blot assays. The results showed that XAB2 knockdown increased the susceptibility of CRC cells to oxaliplatin-induced DNA damage, while XAB2 overexpression alleviated oxaliplatin-induced DNA damage in CRC cells (Fig. 4A-D). Additionally, an alkaline comet assay was performed, revealing that a higher percentage of tail DNA and longer tail moments were associated with severe DNA damage. After 48 h of treatment with oxaliplatin (7.5 µM), XAB2-knockdown cells exhibited longer comet tails and a higher percentage of tail DNA (Fig. 4E). In contrast, XAB2-overexpressing cells exhibited shorter comet tails and a lower percentage of tail DNA (Fig. 4F). These results consistently indicate that XAB2 promotes DNA damage repair and enhances CRC cell resistance to oxaliplatin.

### ANXA2 is identified as a target of XAB2 and mediates XAB2-induced progression in CRC

**Fig. 5:**
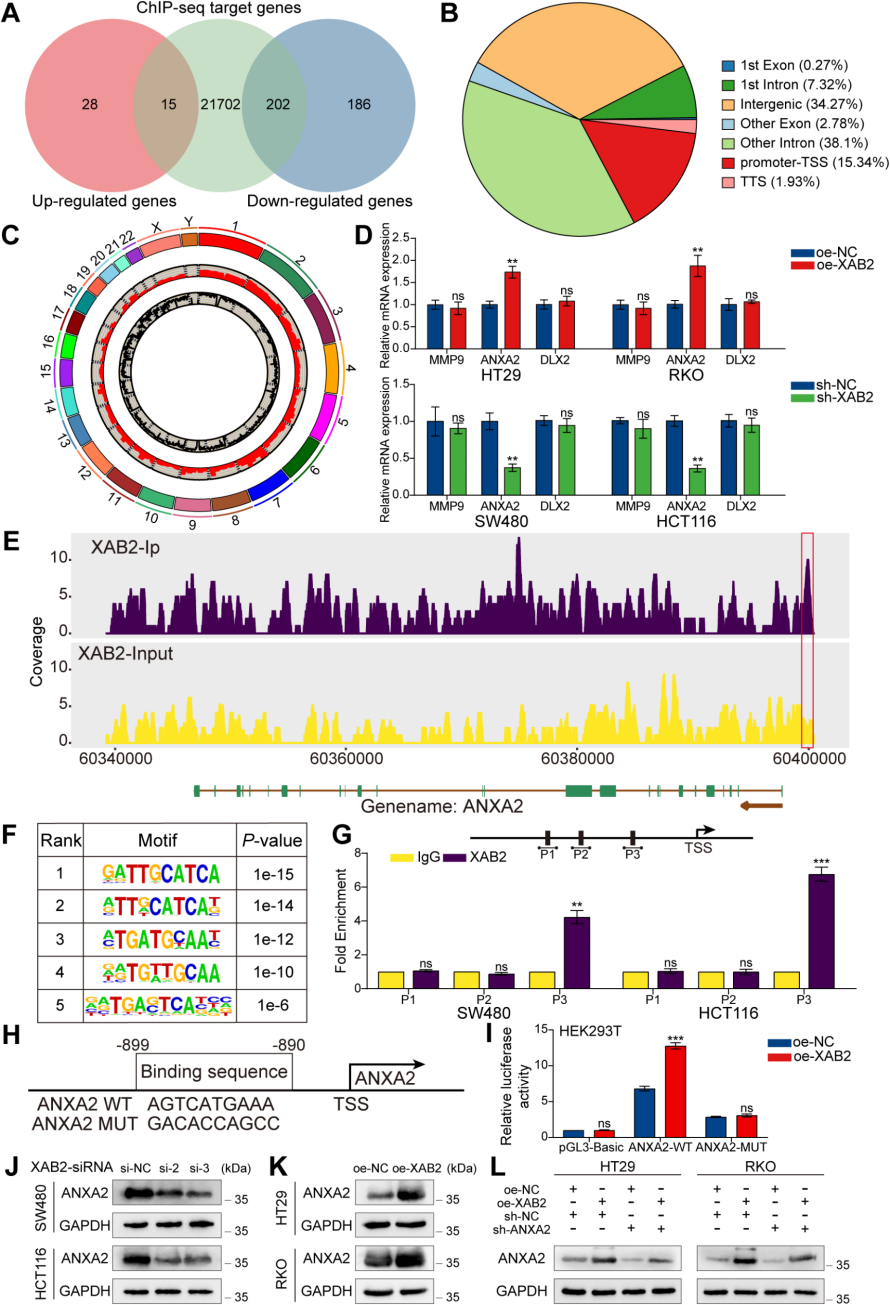
XAB2 binds to the ANXA2 promoter to activate its transcription. **A** Venn diagram illustrating the intersection analysis of RNA-seq and ChIP-seq data. **B** Genome-wide distribution of peaks identified in ChIP-seq data. **C** Chromosomal distribution of reads from ChIP-Seq data. **D** qRT-PCR results showing RNA levels in cells with XAB2 knockdown or overexpression. **E** ChIP-seq peaks showing XAB2 enrichment at the ANXA2 promoter. **F** Five predicted XAB2-binding motifs with the most significant differences among peaks. **G** ChIP-qPCR analysis showing XAB2 enrichment of XAB2 on the ANXA2 promoter relative to control IgG-treated SW480 and HCT116 cell. **H** Putative wild-type and mutant binding sites between XAB2 and the ANXA2 promoter. **I** Dual-luciferase reporter assay using firefly luciferase reporter vectors and Renilla luciferase as an internal control. **J**-**K** Western blot analysis of ANXA2 protein levels in XAB2 overexpressing and knockdown cells. **L** XAB2 plasmid transfected into ANXA2-downregulated CRC cells, with ANXA2 expression confirmed using western blot analysis. Data are presented as mean ± SD (**P < 0.01, ***P < 0.001)

To further elucidate the potential mechanism of XAB2 in CRC, RNA-sequencing (RNA-seq) and Chromatin Immunoprecipitation sequencing (ChIP-seq) were performed to identify potential transcription targets of XAB2. Intersection analysis revealed 217 candidate transcriptional targets directly regulated by XAB2 with 202 genes significantly downregulated in cells with stable XAB2 knockdown (Fig. 5A). ChIP-seq analysis revealed that 15.34% of peaks were located at the promoter-transcription start site (TSS) (Fig. 5B). The distribution of peaks across chromosomes is shown in Fig. 5C. The screened genes were positively regulated by XAB2 and were identified by ChIP-seq as being located at the promoter-TSS. Finally, MMP9, ANXA2, and DLX2—genes that ranked highly in fold change and have been reported to be associated with DNA damage repair and drug resistance—were selected as potential transcription targets for XAB2 [22, 32–34]. Among these, ANXA2 was confirmed via qRT-PCR to be positively regulated by XAB2 at the mRNA level, while MMP9 and DLX2 were not regulated by XAB2 at the mRNA level (Fig. 5D). Additionally, ChIP-seq data revealed significant XAB2-binding peaks in the promoter region of ANXA2 (Fig. 5E). Thus, we hypothesised that ANXA2 could be a potential target gene of XAB2. The top five motifs with the most significant differences are shown in Fig. 5F. Comparison of these motifs with the promoter-binding region of ANXA2 suggested that the fourth motif (Fig. 5F) might serve as a potential binding site. Therefore, ChIP-qPCR assay results indicated that position 3 (P3) could be the binding site between XAB2 and the ANXA2 promoter (Fig. 5G). Figure 5H illustrates the predicted wild-type and mutant binding sites between XAB2 and the ANXA2 promoter. The dual-luciferase reporter assay showed that XAB2 overexpression increased reporter luciferase activity, which was diminished upon mutating the ANXA2 promoter-binding site (Fig. 5I). Western blot analysis indicated that XAB2 positively regulates ANXA2 expression at the protein level (Fig. 5J-L). Collectively, these data indicate that XAB2 activates ANXA2 expression by binding to its promoter.

**Fig. 6:**
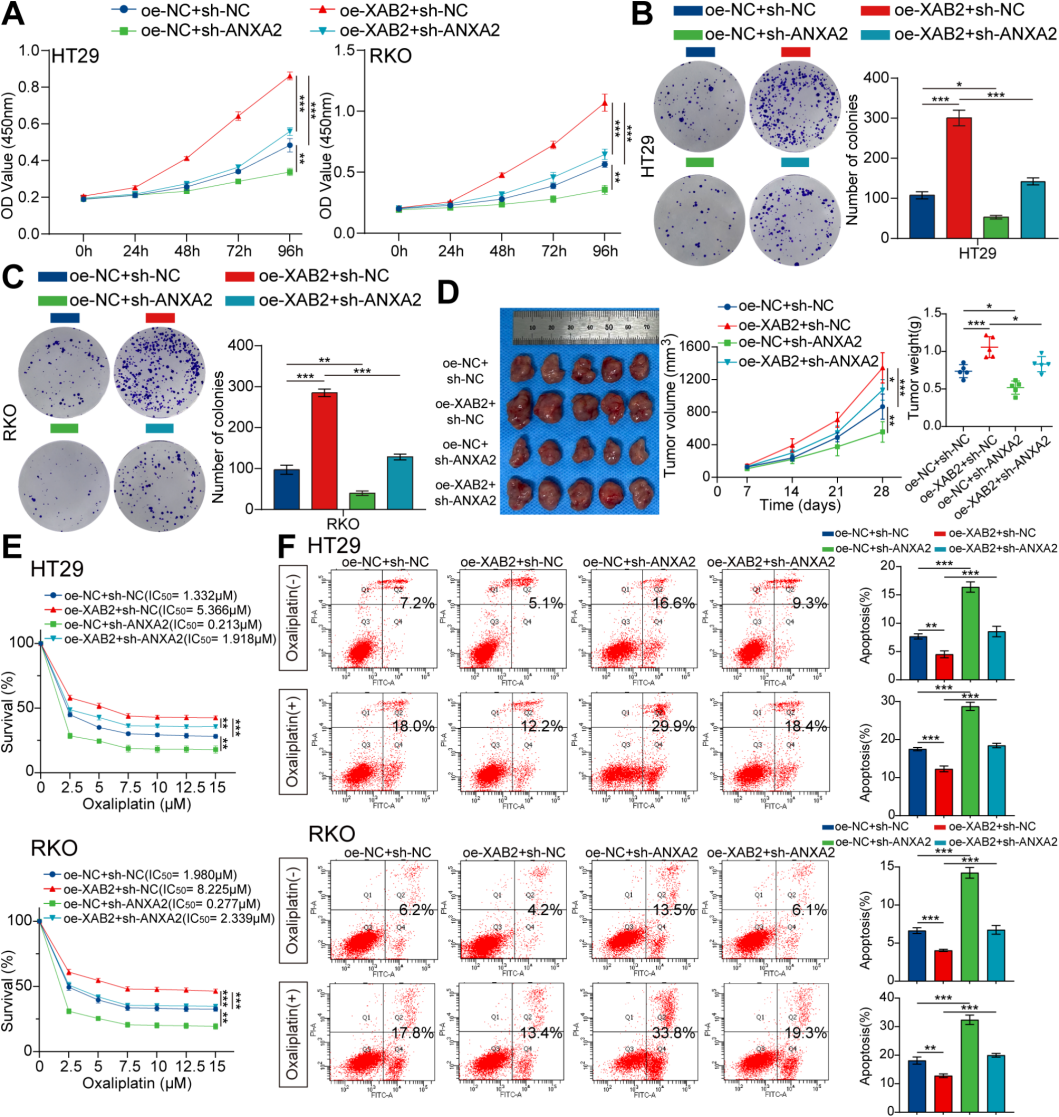
ANXA2 mediates XAB2-induced proliferation and oxaliplatin resistance in CRC cells. **A-C** XAB2 plasmid transfected into ANXA2-downregulated CRC cells, with the proliferative ability of cells determined by CCK8 assay (**A**) and colony formation assay (**B**-**C**). **D** Transplanted xenografts derived from cells with oe-NC + sh-NC, oe-XAB2 + sh-NC, oe-NC + sh-ANXA2, and oe-XAB2 + sh-ANXA2 were established in BALB/c nude mice (*n* = 5). Tumor volume and weight were measured. **E** Viability of HT29 and RKO cells analysed using CCK8 assay after treatment with various concentrations of oxaliplatin for 48 h, with IC50 values displayed. **F** Apoptosis of cells treated with or without oxaliplatin (7.5 µM) for 48 h detected by flow cytometry assays. Data are presented as mean ± SD (*P < 0.05, **P < 0.01, ***P < 0.001)

Next, we evaluated whether XAB2 promoted CRC progression through ANXA2 and whether ANXA2 plays a direct role in modulating oxaliplatin sensitivity. The results of the in vitro and in vivo functional experiments indicated that ANXA2 knockdown reverses the effects of XAB2 overexpression on CRC cell proliferation (Fig. 6A-D). The results of the CCK8 assay and flow cytometry indicated that ANXA2 knockdown reverses the effect of XAB2-induced oxaliplatin resistance in CRC cells, resulting in lower IC50 values and higher apoptotic rates (Fig. 6E-F). The results of the in vivo experiments indicated that knockdown of ANXA2 could increase the oxaliplatin sensitivity of CRC cells (Fig. S3).

**Fig. 7:**
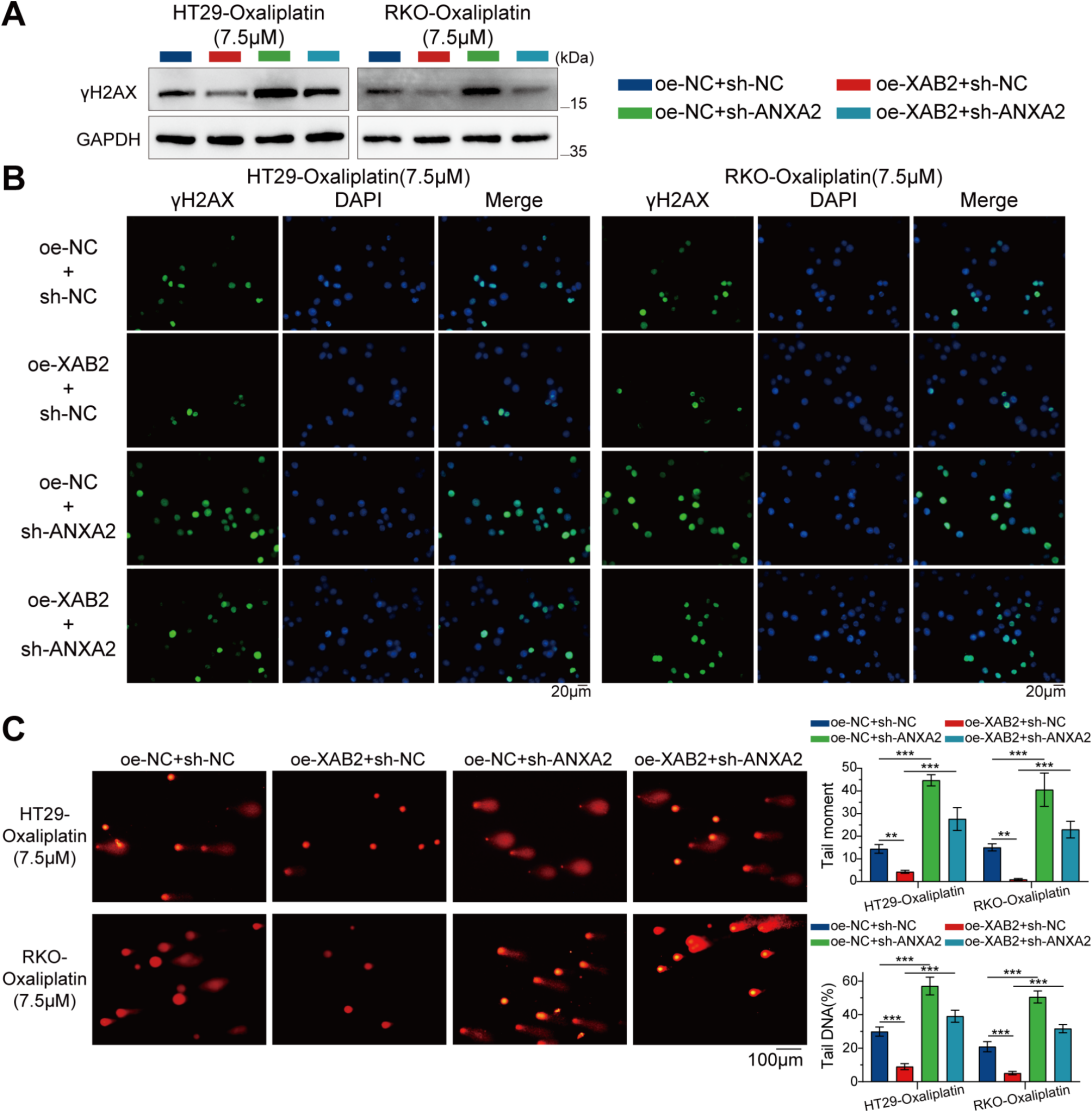
ANXA2 mediates XAB2-induced DNA damage repair in CRC cells. **A** Expression level of γH2AX in CRC cells treated with oxaliplatin (7.5 µM) for 24 h analysed using western blot analysis. **B** γH2AX distribution in CRC cells treated with oxaliplatin (7.5 µM) for 24 h analysed using IF. γH2AX is stained green, and the nucleus is stained blue. Scale bar = 20 μm. **C** Representative images (left) and bar charts (right) from alkaline comet assays of oe-NC + sh-NC, oe-XAB2 + sh-NC, oe-NC + sh-ANXA2, and oe-XAB2 + sh-ANXA2 cells treated with oxaliplatin (7.5 µM) for 48 h. Scale bar = 100 μm. Data are presented as mean ± SD (**P < 0.01, ***P < 0.001)

The level of γH2AX was assessed using western blot and IF assays. The results indicated that ANXA2 knockdown reversed the reduction in oxaliplatin-induced DNA damage caused by XAB2 overexpression (Fig. 7A-B). Moreover, an alkaline comet assay was performed in CRC cells after 48 h of exposure to oxaliplatin (7.5 µM). Notably, ANXA2 knockdown significantly reversed the DNA repair effect induced by XAB2, with longer comet tails and a higher percentage of tail DNA observed in ANXA2-knockdown cells (Fig. 7C). These findings suggest that ANXA2 mediates XAB2 to promote CRC proliferation and DNA damage repair, thereby increasing CRC cell resistance to oxaliplatin.

### USP10 interacts with XAB2 and stabilises its protein expression

**Fig. 8:**
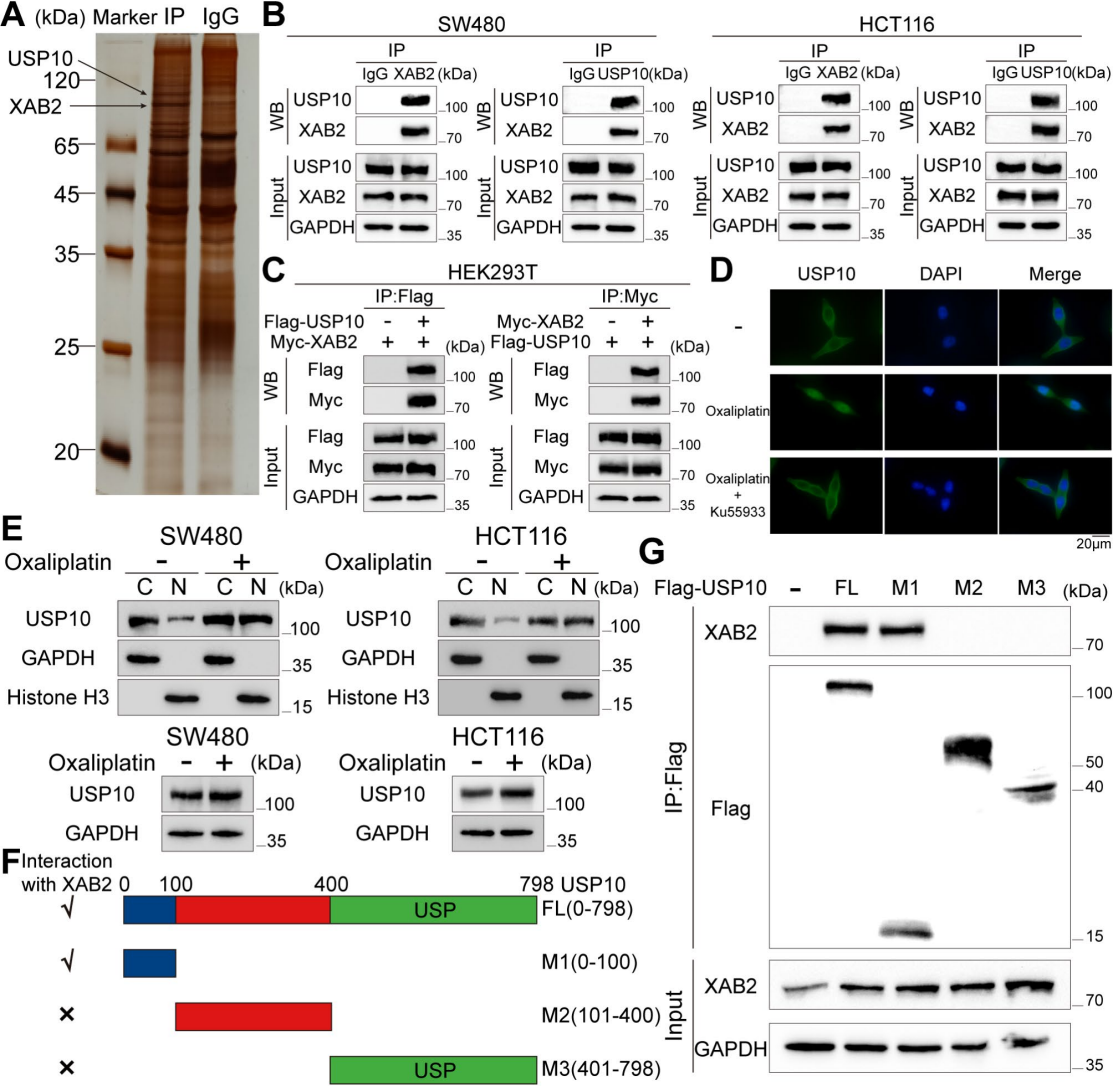
USP10 interacts with XAB2. **A** Distinct bands stained with silver and identified by MS. **B** Lysates from SW480 and HCT116 cells immunoprecipitated with the IgG control, anti-XAB2 antibody, or anti-USP10 antibody followed by immunoblotting with USP10 and XAB2 antibodies. **C** Interaction between Flag-USP10 and Myc-XAB2 confirmed by Co-IP in HEK293T cells. **D** Increase in nuclear expression of USP10 in CRC cells verified by IF after treatment with oxaliplatin (7.5 µM) for 24 h, which can be inhibited by the ATM inhibitor Ku55933 (20mM). **E** Increase in nuclear expression of USP10 in CRC cells verified by western blot analysis after treatment with oxaliplatin (7.5 µM) for 24 h. **F** Schematic representation of Flag-tagged full-length (FL) USP10 with its various deletion mutants. **G** Co-IP confirming the interaction between Myc-XAB2 and Flag-tagged FL USP10 or its indicated mutants in HEK293T cells

Ubiquitination and deubiquitination play critical roles in the DNA damage response [35, 36]. Several proteins of the USP family, including USP3, USP7, USP37, and USP39, are involved in DDR [37–40]. However, the role of ubiquitination and deubiquitination in XAB2 regulation remains unclear. Therefore, mass spectrometry (MS) was performed to identify potential molecules interacting with XAB2, and the results indicated that USP10 may interact with XAB2 (Fig. 8A). Next, co-immunoprecipitation (Co-IP) assays were performed on SW480, HCT116, and HEK-293T cells, confirming that USP10 binds to XAB2 (Fig. 8B-C). Platinum based-drugs, as DNA damage inducers, can activate ATM kinase activity, thereby causing downstream protein nuclear translocation [41, 42]. Previous studies have demonstrated that USP10 translocates into the nucleus following DNA damage, stabilizing nuclear protein expression [43, 44]. Therefore, after treating CRC cells with oxaliplatin (7.5 µM) for 24 h, an IF assay was performed, and nuclear, cytoplasmic, and total proteins were extracted for western blot assay to determine whether oxaliplatin induces this change in USP10 by activating ATM kinase activity. The results indicated that on the one hand, oxaliplatin induced ATM kinase activity, leading to USP10 nuclear translocation, which can be inhibited by the ATM inhibitor Ku55933. On the other hand, oxaliplatin slightly increased the total expression of USP10. These factors collectively led to an increase in nuclear expression of USP10 after exposure to oxaliplatin (Fig. 8D-E). In addition, to identify the structural domain of USP10 that mediates its interaction with XAB2, truncated mutant fragments of USP10 were generated, including mutant 1 (M1) with amino acids 1-100 of USP10, mutant 2 (M2) with amino acids 101–400 of USP10, and mutant 3 (M3) with amino acids 401–798 of USP10 (Fig. 8F). Co-IP assays demonstrated that the amino-terminal region (amino acids 1-100) of USP10 mediated the physical interaction with XAB2, whereas the amino acids 101–400 or amino acids 401–798 did not have this effect (Fig. 8G).

**Fig. 9:**
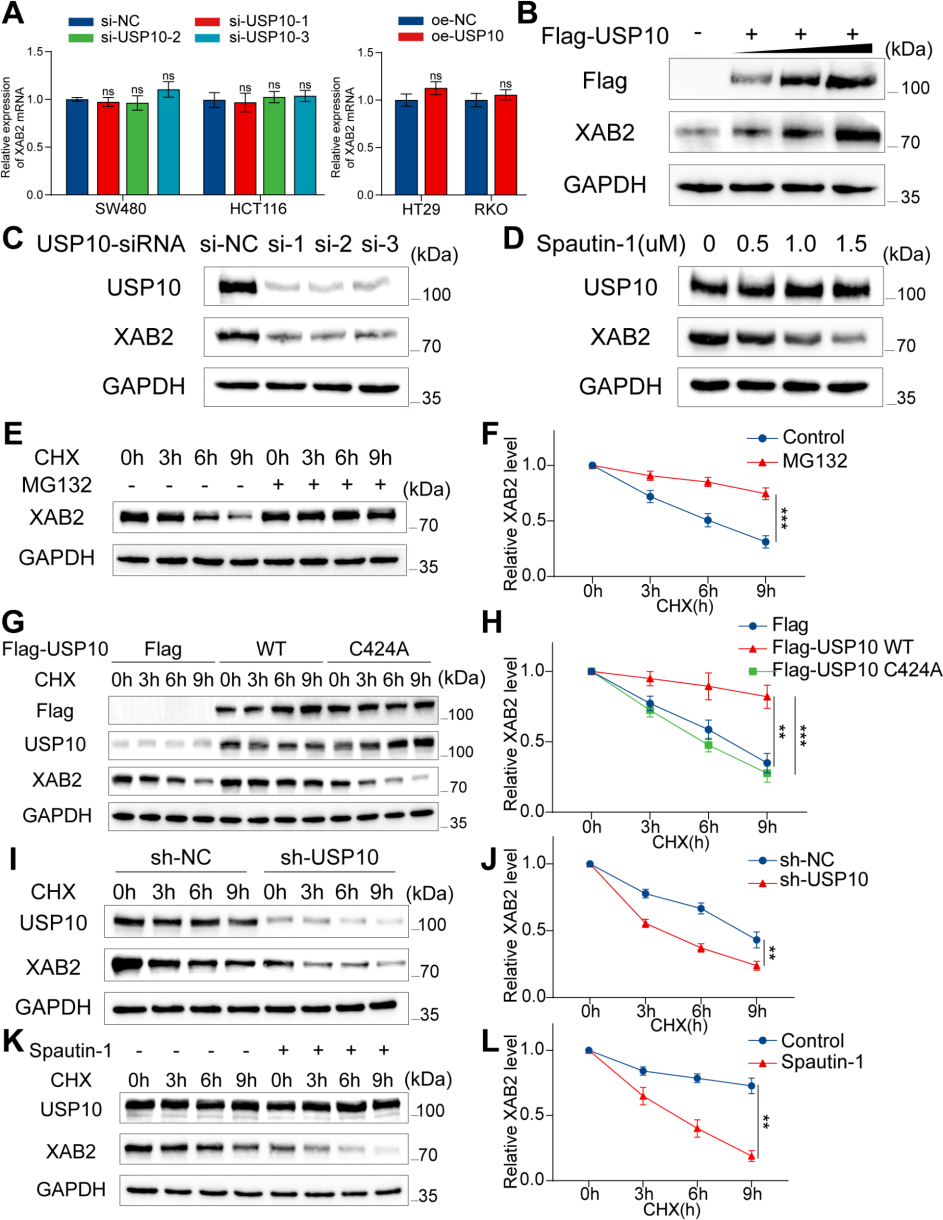
USP10 stabilises XAB2 expression. **A** qRT-PCR analysis indicated that USP10 does not regulate XAB2 at the mRNA level. **B** Western blot analysis demonstrated a gradual increase in XAB2 protein levels with increasing amounts of Flag-USP10 plasmids transfected. **C** Effect of USP10-siRNAs on XAB2 expression 48 h post-transfection. **D** Western blot analysis of XAB2 expression with or without spautin-1 treatment for 24 h at various concentrations. **E**-**F** XAB2 expression levels after varying durations of CHX (50 µg/ml) administration, with or without MG132 (10µM) treatment for 8 h. **G**-**H** XAB2 expression levels after varying durations of CHX (50 µg/ml) administration in cells 48 h post-transfection with Flag vector, Flag-USP10 WT, or Flag-USP10 C424A mutant plasmids. **I**-**J** XAB2 expression levels after varying durations of CHX (50 µg/ml) administration, with or without stable USP10 knockdown. **K**-**L** XAB2 expression levels after varying durations of CHX (50 µg/ml) administration, with or without spautin-1 (1µM) pretreatment. Data are presented as mean ± SD (**P < 0.01, ***P < 0.001)

USP10 deubiquitinates substrates and maintains their stability. To verify whether USP10 can stabilize XAB2, qRT-PCR and western blot analyses were performed, revealing that USP10 does not regulate XAB2 at the mRNA level, while XAB2 protein levels gradually increased with higher amounts of transfected USP10 plasmids (Fig. 9A-B). Consistently, the XAB2 protein levels decreased upon USP10 knockdown (Fig. 9C). To investigate whether this effect depends on USP10 deubiquitination activity, CRC cells were treated with spautin-1, a specific inhibitor of USP10 deubiquitination, for 24 h. The results indicated that spautin-1 decreased XAB2 levels in a concentration-dependent manner (Fig. 9D). The half-life of XAB2 protein under cycloheximide (CHX) treatment was prolonged by the proteasome inhibitor MG132, suggesting that the ubiquitin-proteasome pathway plays a crucial role in XAB2 protein degradation (Fig. 9E-F). Furthermore, overexpression of USP10-wild type (WT) prolonged the half-life of the XAB2 protein, whereas overexpression of USP10-C424A mutant did not (Fig. 9G-H). Conversely, stable knockdown of USP10 or treatment with spautin-1 shortened the half-life of the XAB2 protein (Fig. 9I-L). These results indicate that the upregulation of XAB2 by USP10 depends on the deubiquitination activity of USP10.

### USP10 deubiquitinates XAB2 K48-linked polyubiquitination at K593

**Fig. 10:**
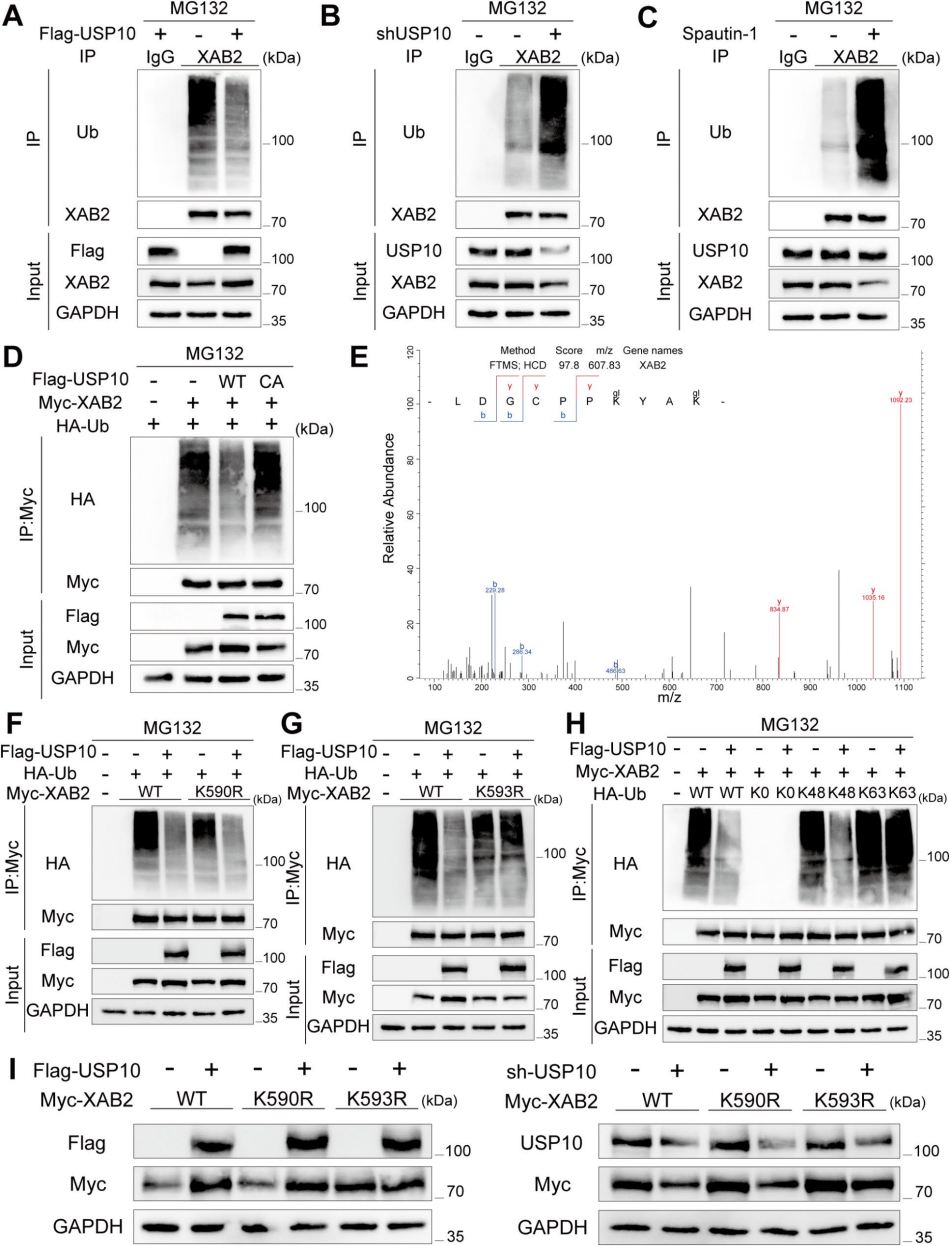
USP10 deubiquitinates XAB2 K48-linked polyubiquitination at K593. **A** SW480 cells lysed 48 h post-transfection with or without Flag-USP10, immunoprecipitated with an anti-XAB2 antibody or IgG control, and subjected to immunoblotting with a ubiquitin (Ub) antibody. **B** Lysates of SW480 cells with or without stable USP10 knockdown immunoprecipitated with an anti-XAB2 antibody or IgG control, followed by immunoblotting with a Ub antibody. **C** The lysates of SW480 cells with or without spautin-1 (1µM) pretreatment for 24 h were immunoprecipitated with the anti-XAB2 antibody or IgG control, followed by immunoblotting with the Ub antibody. **D** SW480 cells lysed 48 h post-transfection with Myc-XAB2, HA-Ub, and Flag-vector/Flag-USP10 WT/Flag-USP10 C424A, respectively, then immunoprecipitated with a Myc antibody and immunoblotted with an HA antibody. **E** Ubiquitination site MS of the XAB2 peptide in SW480 cells with stable USP10 knockdown. **F**-**G** Lysates from SW480 cells lysed 48 h after transfection with Myc-XAB2 WT/Myc-XAB2 K590R/Myc-XAB2 K593R, HA-Ub, and Flag-vector/Flag-USP10, immunoprecipitated using Myc antibody, and immunoblotted with an HA antibody. **H** SW480 cells lysed 48 h after transfection with Myc-XAB2, Flag-USP10, and HA-Ub WT/K0/K48/K63, respectively, then immunoprecipitated with a Myc antibody and immunoblotted with an HA antibody. **I** Western blot analysis indicated the positive XAB2 protein regulation by USP10 depended on the K593 of XAB2

To investigate the effect of USP10 on XAB2 ubiquitination, first, Flag-USP10 plasmid was transfected into CRC cells, resulting in XAB2 deubiquitination (Fig. 10A). Conversely, stable knockdown of USP10 or treatment with spautin-1 increased the ubiquitination of XAB2 (Fig. 10B-C). Additionally, overexpression of the USP10 deubiquitinase inactivation mutant plasmid, USP10-C424A, did not deubiquitinate XAB2 (Fig. 10D). Next, ubiquitination site MS was performed to identify the specific XAB2 deubiquitination sites mediated by USP10. The results indicated that K590 and K593 of XAB2 were potential ubiquitination sites (Fig. 10E). Further experiments revealed that USP10 deubiquitinates K590R but not K593R, identifying K593 as the specific USP10 deubiquitination site for XAB2 (Fig. 10F-G). Different HA-Ub plasmids (WT, K0, K48, and K63), along with Flag-USP10 and Myc-XAB2, were transfected. The results indicated that USP10 specifically removed the K48-linked polyubiquitination of XAB2 (Fig. 10H). Finally, western blot analysis showed that the positive regulation of XAB2 protein by USP10 depended on K593 of XAB2 (Fig. 10I).

### USP10 promotes CRC proliferation, oxaliplatin resistance, and DNA damage repair by stabilising XAB2

**Fig. 11:**
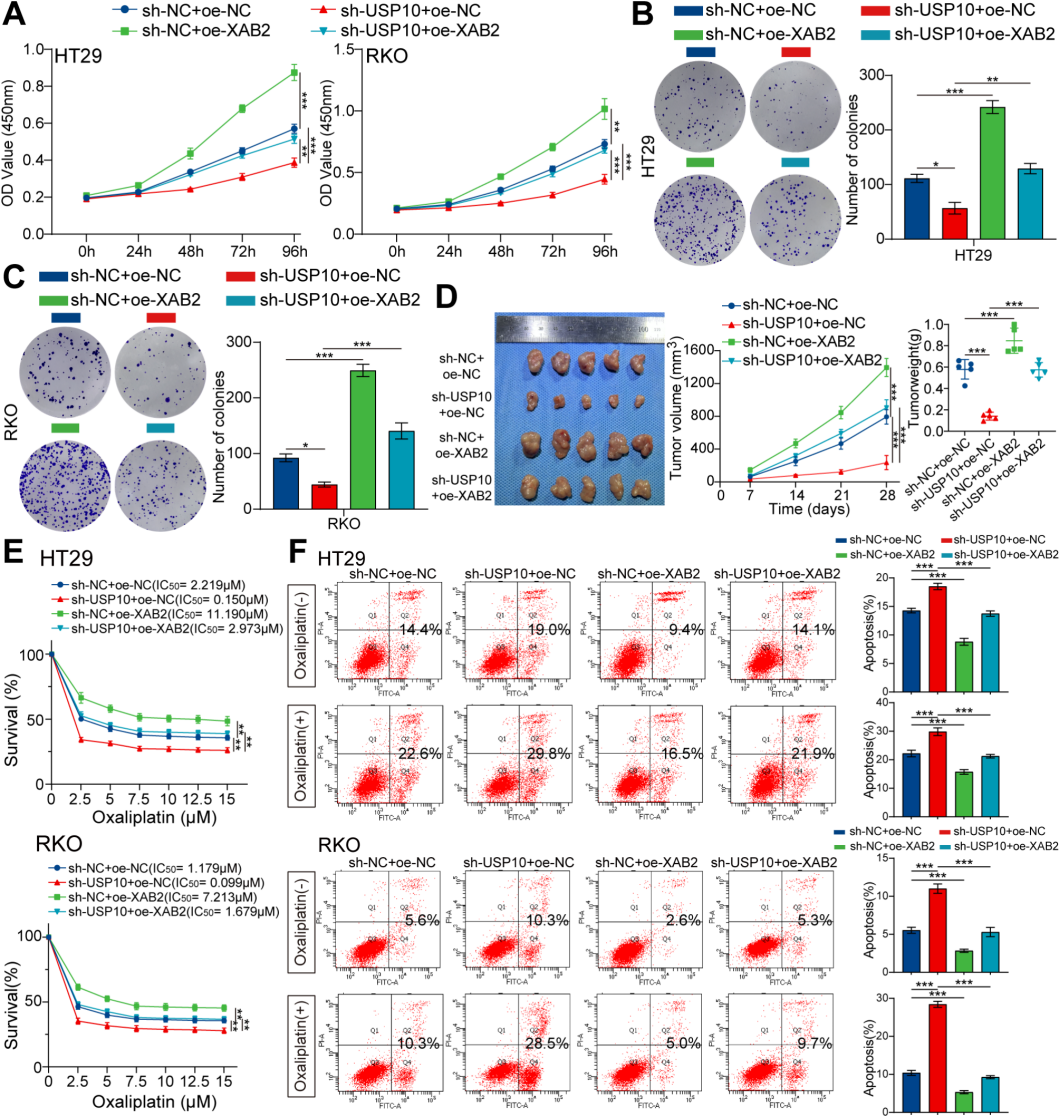
USP10 promotes CRC proliferation and oxaliplatin resistance through XAB2. **A-C** XAB2 plasmid transfected into USP10-downregulated CRC cells, with the proliferative ability of cells assessed using CCK8 assay (**A**) and colony formation assay (**B**-**C**). **D** Transplanted xenografts derived from cells with sh-NC + oe-NC, sh-USP10 + oe-NC, sh-NC + oe-XAB2, and sh-USP10 + oe-XAB2 were established in BALB/c nude mice (*n* = 5). Tumor volume and weight were measured. **E** Viability of HT29 and RKO cells analysed using CCK8 assay after treatment with various concentrations of oxaliplatin for 48 h, with IC50 values displayed. **F** Apoptosis of cells treated with or without oxaliplatin (7.5 µM) for 48 h detected by flow cytometry assays. Data are presented as mean ± SD (*P < 0.05, **P < 0.01, ***P < 0.001)

To assess the functional implications of USP10-mediated regulation of XAB2 in CRC phenotypes, we stably knocked down USP10 in HT29 and RKO cells. USP10 knockdown decreased the proliferation of CRC cells in vitro, an effect that was reversed by the XAB2 (Fig. 11A-C). Consistently, USP10 knockdown impeded the growth of HT29 xenografts, which was reversed by XAB2 overexpression (Fig. 11D). Next, we detected oxaliplatin resistance in CRC cells using CCK8 assays and flow cytometry. The results demonstrated that USP10 knockdown reduced oxaliplatin resistance in CRC cells, resulting in lower IC50 values and higher apoptotic rates, effects reversed by XAB2 overexpression (Fig. 11E-G).

**Fig. 12:**
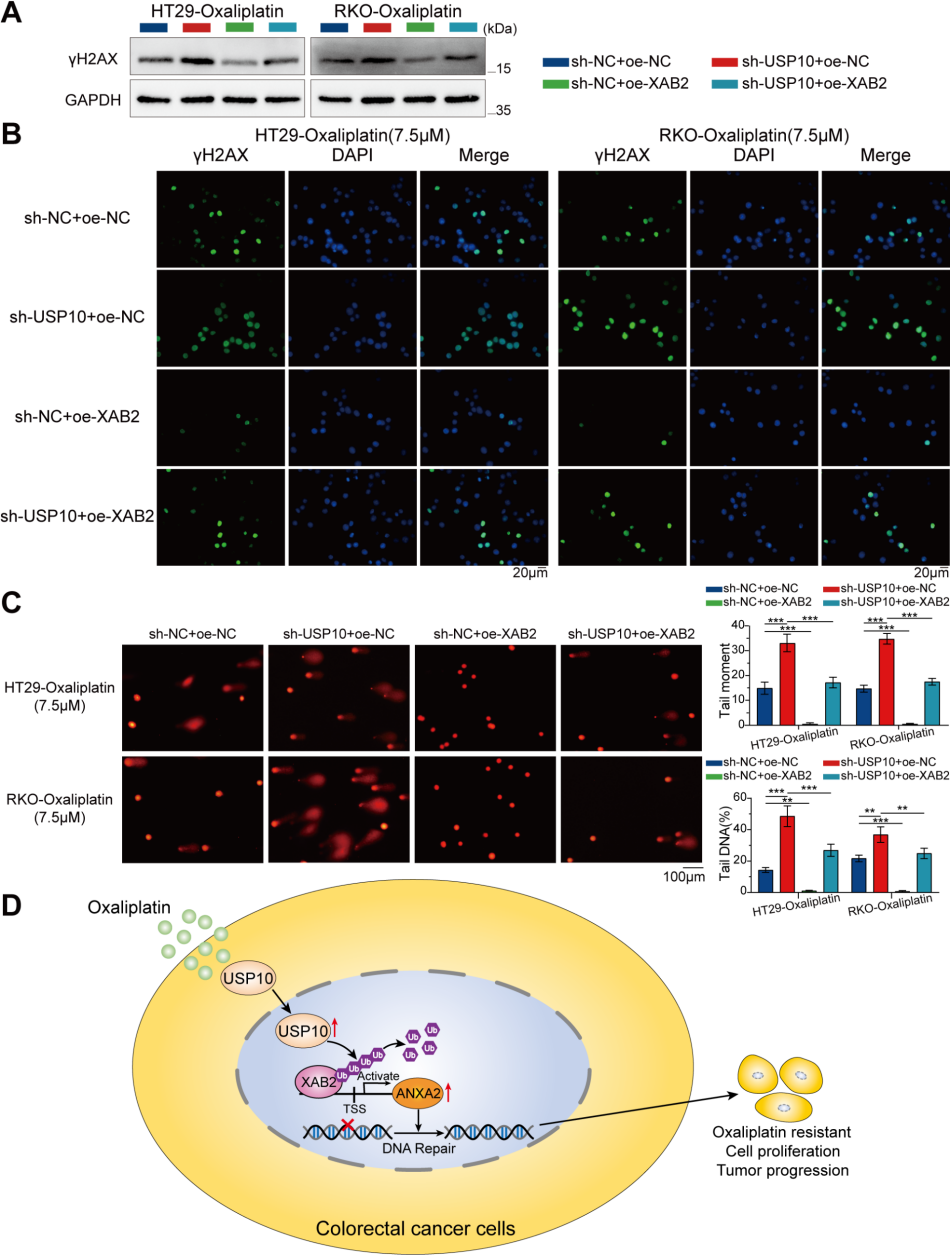
USP10 promotes DNA damage repair in CRC cells through XAB2. **A** Expression level of γH2AX in CRC cells treated with oxaliplatin (7.5 µM) for 24 h analysed using western blot analysis. **B** γH2AX distribution in CRC cells treated with oxaliplatin (7.5 µM) for 24 h analysed using IF. γH2AX is stained green, and the nucleus is stained blue. Scale bar = 20 μm. **C** Representative images (left) and bar charts (right) of alkaline comet assays of sh-NC + oe-NC, sh-USP10 + oe-NC, sh-NC + oe-XAB2, and sh-USP10 + oe-XAB2 cells treated with oxaliplatin (7.5 µM) for 48 h. Scale bar = 100 μm. Data are presented as mean ± SD (**P < 0.01, ***P < 0.001)

γH2AX levels were measured using western blot and IF assays. The results indicated that USP10 knockdown increased oxaliplatin-induced DNA damage, an effect reversed by XAB2 overexpression (Fig. 12A-B). Alkaline comet assay conducted in CRC cells after 48 h of oxaliplatin exposure (7.5 µM) showed that USP10 knockdown significantly decreased DNA repair efficacy, with longer comet tails and higher tail DNA percentage observed in USP10-knockdown cells (Fig. 12C). This effect was reversed upon XAB2 overexpression. Overall, these findings suggest that USP10 promotes cell proliferation, oxaliplatin resistance, and DNA damage repair in CRC by stabilising XAB2.

Collectively, these findings indicate that oxaliplatin treatment increases USP10 nuclear expression in CRC cells, enabling XAB2 deubiquitination and stabilising its protein expression. XAB2 then binds to the ANXA2 promoter to activate its transcriptional activity, ultimately promoting CRC cell proliferation, oxaliplatin resistance, and DNA damage repair (Fig. 12D).

## Discussion

Oxaliplatin is a third-generation platinum-based drug commonly used in the treatment of CRC. It exerts anticancer effects by forming platinum-DNA adducts, which induce DNA damage and apoptosis [45]. However, some patients exhibit inherent or acquired resistance to oxaliplatin, reducing its anticancer efficacy. Elucidating the mechanisms behind oxaliplatin resistance and developing more effective strategies to combat cancer is imperative. In this study, we found that oxaliplatin induces increased nuclear expression of USP10 in CRC cells, which deubiquitinates XAB2 to stabilise its protein. XAB2 binds to the ANXA2 promoter, activating its transcriptional activity, thereby promoting CRC cell proliferation and DNA damage response, repairing oxaliplatin-induced DNA damage, and enhancing oxaliplatin resistance. Therefore, targeting the USP10/XAB2/ANXA2 axis to alleviate oxaliplatin resistance in CRC cells could be a promising therapeutic strategy.

XAB2 regulates various cellular processes, including cell cycle, cell senescence, and DDR [7, 46]. Notably, XAB2 is involved in various DNA damage repair mechanisms, such as promoting the eviction of the non-homologous end-joining factor Ku from single-ended DNA double-strand breaks, which is crucial for homologous recombination [47]. Additionally, XAB2 detaches from R-loops after DNA damage induction, facilitating DNA damage recognition during transcription-coupled nucleotide excision repair [9]. Nucleotide excision repair (NER) has been described as the primary mechanism for repairing oxaliplatin-induced DNA damage [48]. ERCC1, a key mediator in NER, and its catalytic partner, XPF, have been linked to oxaliplatin resistance [49]. Interestingly, XAB2 has been shown to interact with ERCC1-XPF in R-loop processing, supporting its role in oxaliplatin resistance [6]. In addition, genomic instability from DDR defects can drive cancer development. Thus, we investigated whether XAB2 influences CRC initiation and progression. This study is the first to systematically examine the role of XAB2 in the malignant phenotype of CRC cells. Our findings show that XAB2 is highly expressed in CRC, significantly promoting CRC cell proliferation, enhancing DDR, repairing oxaliplatin-induced DNA damage, and increasing oxaliplatin resistance. Additionally, high XAB2 expression was strongly associated with unfavourable clinicopathological features and poor prognosis in patients with CRC.

Previous studies have shown that XAB2 interacts with gene promoters to regulate their transcription [46]. In this study, by combining RNA-seq and ChIP-seq data, we confirmed that ANXA2 was a direct target of XAB2. ANXA2 is an emerging tumor biomarker that promotes cancer progression and is linked to poor prognosis. In CRC, ANXA2 accumulates in the cell nucleus, disrupting coilin and causing its abnormal localisation to the centromere, leading to chromosomal instability. This chromosomal instability accelerates tumor growth and contributes to chemotherapy resistance [50, 51]. In hepatocellular carcinoma (HCC), long non-coding RNA binds to ANXA2, enhances its protein stability, and promotes oxaliplatin resistance [52]. These findings indicate that ANXA2 is a potential target for alleviating resistance to chemotherapy. In this study, the results of in vivo experiments showed that ANXA2 can directly enhance the resistance of CRC cells to oxaliplatin, further confirming these conclusions.

Deubiquitinases mediate substrate protein deubiquitination and regulate their functions. During tumor initiation and progression, deubiquitinases regulate key tumor-related proteins, influencing cell proliferation, apoptosis, DNA damage repair, and other phenotypes. Previous studies revealed a strong link between USP10 and tumor biology. USP10 functions as a tumor suppressor in lung cancer and as an oncogene in several other cancers, including CRC, HCC, and gastric cancer [53–56]. USP10 is also closely associated with DDR. USP10 deubiquitinates PARP1 K48-linked polyubiquitination at K425, preventing its degradation and promoting homologous recombination-mediated DNA double-strand break (DSB) repair to mitigate DNA damage [57]. In addition, USP10 translocates to the nucleus after DNA damage, where it deubiquitinates nuclear substrates [44]. This is similar to the findings of this study, which showed that oxaliplatin treatment increased the nuclear expression of USP10 in CRC cells, leading to the deubiquitination of XAB2 and preventing its degradation. These findings suggest that USP10 influences DDR, thereby impacting tumor progression.

Despite the findings in our study, there are still limitations and questions that warrant further exploration. To investigate the role of XAB2 in oxaliplatin resistance, we used an oxaliplatin-resistant cell line. However, human tissues with or without oxaliplatin resistance should also be used. In practice, postoperative CRC patients typically do not receive oxaliplatin as a monotherapy but instead are treated with combination chemotherapy, including drugs such as capecitabine or 5-fluorouracil. This makes it challenging to assess XAB2-mediated resistance to oxaliplatin monotherapy at the patient tissue level. Another topic worth investigating is that USP10 contains a USP domain at its C-terminus, which typically functions as the deubiquitinating enzyme activity domain. However, the amino-terminal region (amino acids 1-100) of USP10 was found to mediate the interaction with XAB2. There are several potential mechanisms behind this phenomenon, including: (1) The N-terminal region of USP10 could serve as a binding platform that recruits XAB2 to USP10; (2) The binding of XAB2 to the N-terminal region of USP10 might induce a conformational change in USP10; (3) The N-terminal region of USP10 might recruit additional cofactors or adaptor proteins that facilitate the deubiquitylation of XAB2; (4) The N-terminal region of USP10 might help expose or remodel the ubiquitin chains on XAB2, making them more accessible to the USP domain. In summary, the N-terminal region of USP10 plays a critical role in recruiting XAB2 and positioning it in a way that allows the USP domain to deubiquitylate it, even without direct binding. However, the specific mechanism remains unclear, and further research is needed to elucidate it.

## Conclusions

In summary, our findings reveal that XAB2 is highly expressed in CRC and correlates with poor prognosis. XAB2 directly binds to the ANXA2 promoter and regulates its transcriptional activity, thereby regulating cell proliferation, DNA damage repair, and oxaliplatin resistance in CRC. Additionally, we demonstrated that this regulatory mechanism is modulated by USP10 through XAB2 ubiquitination. Oxaliplatin treatment increases USP10 nuclear expression, enabling it to deubiquitinate XAB2 K48-linked polyubiquitination at K593, thereby stabilising XAB2 by preventing its degradation via the ubiquitin-proteasome pathway. This study highlights the potential of targeting the USP10/XAB2/ANXA2 axis to enhance CRC sensitivity to oxaliplatin, presenting a promising therapeutic strategy for further investigation.

## Electronic supplementary material

Below is the link to the electronic supplementary material.

Supplementary Material 1: Supplementary Methods

Supplementary Material 2: Table S1: Association between XAB2 expression and clinicopathological parameters in colorectal cancer patients.

Supplementary Material 3: Figure S1: Construction and validation of prognostic model based on XAB2. A Kaplan–Meier analysis showing OS and PFS curves of patients with CRC stratified by high versus low XAB2 expression from Kaplan-Meier plotter website. B Nomogram Predicting OS for patients with CRC in TCGA Cohorts. C The calibration curve of the nomogram. D The time-dependent ROC curve of the nomogram.

Supplementary Material 4: Figure S2: XAB2 enhances the expression of ki-67 and PCNA in vivo. A-B Representative IHC images of ki-67 and PCNA expression in xenograft tumor tissues from the indicated groups.

Supplementary Material 5: Figure S3: ANXA2 increases the oxaliplatin resistance of CRC cells in vivo. Transplanted xenografts derived from cells with sh-NC and sh-ANXA2 were established in BALB/c nude mice (*n* = 5). One week after injection, oxaliplatin (20 mg/kg) or an equivalent volume of saline was injected into the tumor every 5 days for 20 days. Tumor volume and weight were measured. Data are presented as mean ± SD (**P < 0.01, ***P < 0.001).

## Data Availability

No datasets were generated or analysed during the current study.

## Abbreviations

CRC: Colorectal cancer
XAB2: XPA binding protein 2
DDR: DNA damage response
UPS: Ubiquitin-proteasome system
DUBs: Deubiquitinases
USP: Ubiquitin-specific protease
ANXA2: Annexin A2
IHC: Immunohistochemistry
SPSS: Statistical Package for the Social Sciences
SD: Standard deviation
ANOVA: Analysis of variance
COADREAD: Colorectal adenocarcinoma
TCGA: The Cancer Genome Atlas
COAD: Colon adenocarcinoma
READ: Rectal adenocarcinoma
qRT-PCR: Quantitative real-time PCR
OS: Overall survival
PFS: Progression-free survival
ROC: Receiver operating characteristic
AUC: Area under the curve
CCK8: Cell Counting Kit-8
GSEA: Gene set enrichment analysis
IC50: Half-maximal inhibitory concentration
IF: Immunofluorescent
RNA-seq: RNA-sequencing
ChIP-seq: Chromatin Immunoprecipitation sequencing
TSS: Transcription start site
MS: Mass spectrometry
Co-IP: Co-immunoprecipitation
M1: Mutant 1
M2: Mutant 2
M3: Mutant 3
CHX: Cycloheximide
WT: Wild type
NER: Nucleotide excision repair
HCC: Hepatocellular carcinoma
DSB: Double strand break

## References

[1] Bray F, Laversanne M, Sung H, Ferlay J, Siegel RL, Soerjomataram I, et al. Global cancer statistics 2022: GLOBOCAN estimates of incidence and mortality worldwide for 36 cancers in 185 countries. CA Cancer J Clin. 2024;74(3):229–63. 10.3322/caac.21834. 38572751 10.3322/caac.21834

[2] Ciardiello F, Ciardiello D, Martini G, Napolitano S, Tabernero J, Cervantes A. Clinical management of metastatic colorectal cancer in the era of precision medicine. CA Cancer J Clin. 2022;72(4):372–401. 10.3322/caac.21728. 35472088 10.3322/caac.21728

[3] Van Cutsem E, Cervantes A, Adam R, Sobrero A, Van Krieken JH, Aderka D, et al. ESMO consensus guidelines for the management of patients with metastatic colorectal cancer. Ann Oncol. 2016;27(8):1386–422. 10.1093/annonc/mdw235. 27380959 10.1093/annonc/mdw235

[4] Ruiz de Porras V, Bystrup S, Martínez-Cardús A, Pluvinet R, Sumoy L, Howells L, et al. Curcumin mediates oxaliplatin-acquired resistance reversion in colorectal cancer cell lines through modulation of CXC-Chemokine/NF-κB signalling pathway. Sci Rep. 2016;6:24675. 10.1038/srep24675. 27091625 10.1038/srep24675 PMC4835769

[5] Rottenberg S, Disler C, Perego P. The rediscovery of platinum-based cancer therapy. Nat Rev Cancer. 2021;21(1):37–50. 10.1038/s41568-020-00308-y. 33128031 10.1038/s41568-020-00308-y

[6] Goulielmaki E, Tsekrekou M, Batsiotos N, Ascensão-Ferreira M, Ledaki E, Stratigi K, et al. The splicing factor XAB2 interacts with ERCC1-XPF and XPG for R-loop processing. Nat Commun. 2021;12(1):3153. 10.1038/s41467-021-23505-1. 34039990 10.1038/s41467-021-23505-1 PMC8155215

[7] Hou S, Qu D, Li Y, Zhu B, Liang D, Wei X, et al. XAB2 depletion induces intron retention in POLR2A to impair global transcription and promote cellular senescence. Nucleic Acids Res. 2019;47(15):8239–54. 10.1093/nar/gkz532. 31216022 10.1093/nar/gkz532 PMC6735682

[8] Onyango DO, Howard SM, Neherin K, Yanez DA, Stark JM. Tetratricopeptide repeat factor XAB2 mediates the end resection step of homologous recombination. Nucleic Acids Res. 2016;44(12):5702–16. 10.1093/nar/gkw275. 27084940 10.1093/nar/gkw275 PMC4937314

[9] Donnio L-M, Cerutti E, Magnani C, Neuillet D, Mari P-O, Giglia-Mari G. XAB2 dynamics during DNA damage-dependent transcription Inhibition. Elife. 2022;11. 10.7554/eLife.77094. 10.7554/eLife.77094 PMC9436415 35880862

[10] Jackson SP, Bartek J. The DNA-damage response in human biology and disease. Nature. 2009;461(7267):1071–8. 10.1038/nature08467. 19847258 10.1038/nature08467 PMC2906700

[11] Hanahan D, Weinberg RA. Hallmarks of cancer: the next generation. Cell. 2011;144(5):646–74. 10.1016/j.cell.2011.02.013. 21376230 10.1016/j.cell.2011.02.013

[12] Liu F, Chen J, Li K, Li H, Zhu Y, Zhai Y, et al. Ubiquitination and deubiquitination in cancer: from mechanisms to novel therapeutic approaches. Mol Cancer. 2024;23(1):148. 10.1186/s12943-024-02046-3. 39048965 10.1186/s12943-024-02046-3 PMC11270804

[13] Gao H, Yin J, Ji C, Yu X, Xue J, Guan X, et al. Targeting ubiquitin specific proteases (USPs) in cancer immunotherapy: from basic research to preclinical application. J Exp Clin Cancer Res. 2023;42(1):225. 10.1186/s13046-023-02805-y. 37658402 10.1186/s13046-023-02805-y PMC10472646

[14] Li H, Feng H, Zhang T, Wu J, Shen X, Xu S, et al. CircHAS2 activates CCNE2 to promote cell proliferation and sensitizes the response of colorectal cancer to anlotinib. Mol Cancer. 2024;23(1):59. 10.1186/s12943-024-01971-7. 38515149 10.1186/s12943-024-01971-7 PMC10956180

[15] Sun L, Yu J, Guinney J, Qin B, Sinicrope FA. USP10 regulates ZEB1 ubiquitination and protein stability to inhibit ZEB1-Mediated colorectal Cancer metastasis. Mol Cancer Res. 2023;21(6):578–90. 10.1158/1541-7786.MCR-22-0552. 36940483 10.1158/1541-7786.MCR-22-0552 PMC10239320

[16] Reissland M, Hartmann O, Tauch S, Bugter JM, Prieto-Garcia C, Schulte C, et al. USP10 drives cancer stemness and enables super-competitor signalling in colorectal cancer. Oncogene. 2024;43(50):3645–59. 10.1038/s41388-024-03141-x. 39443725 10.1038/s41388-024-03141-x PMC11611742

[17] Wang J, He Z, Liu X, Xu J, Jiang X, Quan G, et al. LINC00941 promotes pancreatic cancer malignancy by interacting with ANXA2 and suppressing NEDD4L-mediated degradation of ANXA2. Cell Death Dis. 2022;13(8):718. 10.1038/s41419-022-05172-2. 35977942 10.1038/s41419-022-05172-2 PMC9385862

[18] Mao L, Yuan W, Cai K, Lai C, Huang C, Xu Y, et al. EphA2-YES1-ANXA2 pathway promotes gastric cancer progression and metastasis. Oncogene. 2021;40(20):3610–23. 10.1038/s41388-021-01786-6. 33941853 10.1038/s41388-021-01786-6 PMC8134040

[19] Ma S, Lu C-C, Yang L-Y, Wang J-J, Wang B-S, Cai H-Q, et al. ANXA2 promotes esophageal cancer progression by activating MYC-HIF1A-VEGF axis. J Exp Clin Cancer Res. 2018;37(1):183. 10.1186/s13046-018-0851-y. 30081903 10.1186/s13046-018-0851-y PMC6091180

[20] Yang T, Peng H, Wang J, Yang J, Nice EC, Xie K, et al. Prognostic and diagnostic significance of Annexin A2 in colorectal cancer. Colorectal Dis. 2013;15(7):e373–81. 10.1111/codi.12207. 23489866 10.1111/codi.12207

[21] Zhou L, Li J, Tang Y, Yang M. Exosomal LncRNA LINC00659 transferred from cancer-associated fibroblasts promotes colorectal cancer cell progression via miR-342-3p/ANXA2 axis. J Transl Med. 2021;19(1):8. 10.1186/s12967-020-02648-7. 33407563 10.1186/s12967-020-02648-7 PMC7789760

[22] Zhou L, Li J, Liao M, Zhang Q, Yang M. LncRNA MIR155HG induces M2 macrophage polarization and drug resistance of colorectal cancer cells by regulating ANXA2. Cancer Immunol Immunother. 2022;71(5):1075–91. 10.1007/s00262-021-03055-7. 34562123 10.1007/s00262-021-03055-7 PMC10991596

[23] Hong W, Ying H, Lin F, Ding R, Wang W, Zhang M. LncRNA LINC00460 Silencing represses EMT in Colon cancer through downregulation of ANXA2 via upregulating miR-433-3p. Mol Ther Nucleic Acids. 2020;19:1209–18. 10.1016/j.omtn.2019.12.006. 32069703 10.1016/j.omtn.2019.12.006 PMC7019044

[24] Hong Y, Downey T, Eu KW, Koh PK, Cheah PY. A ‘metastasis-prone’ signature for early-stage mismatch-repair proficient sporadic colorectal cancer patients and its implications for possible therapeutics. Clin Exp Metastasis. 2010;27(2):83–90. 10.1007/s10585-010-9305-4. 20143136 10.1007/s10585-010-9305-4

[25] Gravett AM, Dennis JL, Dalgleish AG, Copier J, Liu WM. The efficacy of chemotherapeutic drug combinations May be predicted by concordance of gene response to the single agents. Oncol Lett. 2020;20(6):321. 10.3892/ol.2020.12184. 33093925 10.3892/ol.2020.12184 PMC7573875

[26] Marisa L, de Reyniès A, Duval A, Selves J, Gaub MP, Vescovo L, et al. Gene expression classification of colon cancer into molecular subtypes: characterization, validation, and prognostic value. PLoS Med. 2013;10(5):e1001453. 10.1371/journal.pmed.1001453. 23700391 10.1371/journal.pmed.1001453 PMC3660251

[27] An N, Shi X, Zhang Y, Lv N, Feng L, Di X, et al. Discovery of a novel immune gene signature with profound prognostic value in colorectal cancer: A model of cooperativity disorientation created in the process from development to Cancer. PLoS ONE. 2015;10(9):e0137171. 10.1371/journal.pone.0137171. 26325386 10.1371/journal.pone.0137171 PMC4556644

[28] Kagawa Y, Matsumoto S, Kamioka Y, Mimori K, Naito Y, Ishii T, et al. Cell cycle-dependent Rho GTPase activity dynamically regulates cancer cell motility and invasion in vivo. PLoS ONE. 2013;8(12):e83629. 10.1371/journal.pone.0083629. 24386239 10.1371/journal.pone.0083629 PMC3875446

[29] Iwaya T, Yokobori T, Nishida N, Kogo R, Sudo T, Tanaka F, et al. Downregulation of miR-144 is associated with colorectal cancer progression via activation of mTOR signaling pathway. Carcinogenesis. 2012;33(12):2391–7. 10.1093/carcin/bgs288. 22983984 10.1093/carcin/bgs288

[30] Ballout F, Lu H, Bhat N, Chen L, Peng D, Chen Z, et al. Targeting SMAD3 improves response to oxaliplatin in esophageal adenocarcinoma models by impeding DNA repair. Clin Cancer Res. 2024;30(10):2193–205. 10.1158/1078-0432.CCR-24-0027. 38592373 10.1158/1078-0432.CCR-24-0027 PMC11096039

[31] Li Y, Gan Y, Liu J, Li J, Zhou Z, Tian R, et al. Downregulation of MEIS1 mediated by ELFN1-AS1/EZH2/DNMT3a axis promotes tumorigenesis and oxaliplatin resistance in colorectal cancer. Signal Transduct Target Ther. 2022;7(1):87. 10.1038/s41392-022-00902-6. 35351858 10.1038/s41392-022-00902-6 PMC8964798

[32] Walter L, Canup B, Pujada A, Bui TA, Arbasi B, Laroui H, et al. Matrix metalloproteinase 9 (MMP9) limits reactive oxygen species (ROS) accumulation and DNA damage in colitis-associated cancer. Cell Death Dis. 2020;11(9):767. 10.1038/s41419-020-02959-z. 32943603 10.1038/s41419-020-02959-z PMC7498454

[33] Takahashi H, Katsuta E, Yan L, Dasgupta S, Takabe K. High expression of Annexin A2 is associated with DNA repair, metabolic alteration, and worse survival in pancreatic ductal adenocarcinoma. Surgery. 2019;166(2):150–6. 10.1016/j.surg.2019.04.011. 31171367 10.1016/j.surg.2019.04.011 PMC6661011

[34] Zhang B, Du X, Fan Y, Qu G, Pang LK, Zhao R, et al. DLX2 promotes osteosarcoma epithelial-mesenchymal transition and doxorubicin resistance by enhancing HOXC8-CDH2 axis. iScience. 2023;26(11):108272. 10.1016/j.isci.2023.108272. 38026218 10.1016/j.isci.2023.108272 PMC10651674

[35] Jackson SP, Durocher D. Regulation of DNA damage responses by ubiquitin and SUMO. Mol Cell. 2013;49(5):795–807. 10.1016/j.molcel.2013.01.017. 23416108 10.1016/j.molcel.2013.01.017

[36] Le J, Perez E, Nemzow L, Gong F. Role of deubiquitinases in DNA damage response. DNA Repair (Amst). 2019;76:89–98. 10.1016/j.dnarep.2019.02.011. 30831436 10.1016/j.dnarep.2019.02.011 PMC6561784

[37] Li S, Xiong S, Li Z, Yang L, Yang H, Xiong J, et al. USP3 promotes DNA damage response and chemotherapy resistance through stabilizing and deubiquitinating SMARCA5 in prostate cancer. Cell Death Dis. 2024;15(11):790. 10.1038/s41419-024-07117-3. 39500888 10.1038/s41419-024-07117-3 PMC11538284

[38] Su D, Ma S, Shan L, Wang Y, Wang Y, Cao C, et al. Ubiquitin-specific protease 7 sustains DNA damage response and promotes cervical carcinogenesis. J Clin Invest. 2018;128(10):4280–96. 10.1172/JCI120518. 30179224 10.1172/JCI120518 PMC6159995

[39] Wu C, Chang Y, Chen J, Su Y, Li L, Chen Y, et al. USP37 regulates DNA damage response through stabilizing and deubiquitinating BLM. Nucleic Acids Res. 2021;49(19):11224–40. 10.1093/nar/gkab842. 34606619 10.1093/nar/gkab842 PMC8565321

[40] Wu J, Chen Y, Geng G, Li L, Yin P, Nowsheen S, et al. USP39 regulates DNA damage response and chemo-radiation resistance by deubiquitinating and stabilizing CHK2. Cancer Lett. 2019;449:114–24. 10.1016/j.canlet.2019.02.015. 30771428 10.1016/j.canlet.2019.02.015

[41] Chen J-H, Zhang P, Chen W-D, Li D-D, Wu X-Q, Deng R, et al. ATM-mediated PTEN phosphorylation promotes PTEN nuclear translocation and autophagy in response to DNA-damaging agents in cancer cells. Autophagy. 2015;11(2):239–52. 10.1080/15548627.2015.1009767. 25701194 10.1080/15548627.2015.1009767 PMC4502816

[42] Sigala I, Koutroumani M, Koukiali A, Giannakouros T, Nikolakaki E. Nuclear translocation of SRPKs is associated with 5-FU and cisplatin sensitivity in HeLa and T24 cells. Cells. 2021;10(4). 10.3390/cells10040759. 10.3390/cells10040759 PMC8065462 33808326

[43] Luo Y, Zhang X, Chen R, Li R, Liu Y, Zhang J, et al. USP10 regulates B cell response to SARS-CoV-2 or HIV-1 nanoparticle vaccines through deubiquitinating AID. Signal Transduct Target Ther. 2022;7(1):7. 10.1038/s41392-021-00858-z. 34983926 10.1038/s41392-021-00858-z PMC8724756

[44] Yuan J, Luo K, Zhang L, Cheville JC, Lou Z. USP10 regulates p53 localization and stability by deubiquitinating p53. Cell. 2010;140(3):384–96. 10.1016/j.cell.2009.12.032. 20096447 10.1016/j.cell.2009.12.032 PMC2820153

[45] Faivre S, Chan D, Salinas R, Woynarowska B, Woynarowski JM. DNA strand breaks and apoptosis induced by oxaliplatin in cancer cells. Biochem Pharmacol. 2003;66(2):225–37. 12826265 10.1016/s0006-2952(03)00260-0

[46] Hou S, Li N, Zhang Q, Li H, Wei X, Hao T, et al. XAB2 functions in mitotic cell cycle progression via transcriptional regulation of CENPE. Cell Death Dis. 2016;7(10):e2409. 10.1038/cddis.2016.313. 27735937 10.1038/cddis.2016.313 PMC5133980

[47] Sharma AB, Erasimus H, Pinto L, Caron M-C, Gopaul D, Peterlini T, et al. XAB2 promotes Ku eviction from single-ended DNA double-strand breaks independently of the ATM kinase. Nucleic Acids Res. 2021;49(17):9906–25. 10.1093/nar/gkab785. 34500463 10.1093/nar/gkab785 PMC8464071

[48] Martinez-Balibrea E, Martínez-Cardús A, Ginés A, Ruiz de Porras V, Moutinho C, Layos L, et al. Tumor-Related molecular mechanisms of oxaliplatin resistance. Mol Cancer Ther. 2015;14(8):1767–76. 10.1158/1535-7163.MCT-14-0636. 26184483 10.1158/1535-7163.MCT-14-0636

[49] Huang M-Y, Huang Y-J, Cheng T-L, Jhang W-Y, Ke C-C, Chen Y-T, et al. XPF-ERCC1 blocker improves the therapeutic efficacy of 5-FU- and Oxaliplatin-Based chemoradiotherapy in colorectal Cancer. Cells. 2023;12(11). 10.3390/cells12111475. 10.3390/cells12111475 PMC10252687 37296596

[50] Kazami T, Nie H, Satoh M, Kuga T, Matsushita K, Kawasaki N, et al. Nuclear accumulation of Annexin A2 contributes to chromosomal instability by coilin-mediated centromere damage. Oncogene. 2015;34(32):4177–89. 10.1038/onc.2014.345. 25347736 10.1038/onc.2014.345

[51] Bakhoum SF, Compton DA. Chromosomal instability and cancer: a complex relationship with therapeutic potential. J Clin Invest. 2012;122(4):1138–43. 10.1172/JCI59954. 22466654 10.1172/JCI59954 PMC3314464

[52] Huang J, Lin J, Zhong T, Qin Z, Li G, Yi T, et al. LINC00894 targets Annexin A2 to regulate oxaliplatin resistance in hepatocellular carcinoma: ANXA2 protein function. Int J Biol Macromol. 2024;281(Pt 3):136538. 10.1016/j.ijbiomac.2024.136538. 39396585 10.1016/j.ijbiomac.2024.136538

[53] Wang X, Xia S, Li H, Wang X, Li C, Chao Y, et al. The deubiquitinase USP10 regulates KLF4 stability and suppresses lung tumorigenesis. Cell Death Differ. 2020;27(6):1747–64. 10.1038/s41418-019-0458-7. 31748695 10.1038/s41418-019-0458-7 PMC7244734

[54] Li B, Qi Z-P, He D-L, Chen Z-H, Liu J-Y, Wong M-W, et al. NLRP7 deubiquitination by USP10 promotes tumor progression and tumor-associated macrophage polarization in colorectal cancer. J Exp Clin Cancer Res. 2021;40(1):126. 10.1186/s13046-021-01920-y. 33838681 10.1186/s13046-021-01920-y PMC8035766

[55] Lin C, Lin P, Yao H, Liu S, Lin X, He R, et al. Modulation of YBX1-mediated PANoptosis Inhibition by PPM1B and USP10 confers chemoresistance to oxaliplatin in gastric cancer. Cancer Lett. 2024;587:216712. 10.1016/j.canlet.2024.216712. 38364962 10.1016/j.canlet.2024.216712

[56] Zhu H, Yan F, Yuan T, Qian M, Zhou T, Dai X, et al. USP10 promotes proliferation of hepatocellular carcinoma by deubiquitinating and stabilizing YAP/TAZ. Cancer Res. 2020;80(11):2204–16. 10.1158/0008-5472.CAN-19-2388. 32217697 10.1158/0008-5472.CAN-19-2388

[57] Zhao X, Ma Y, Li J, Sun X, Sun Y, Qu F, et al. The AEG-1-USP10-PARP1 axis confers radioresistance in esophageal squamous cell carcinoma via facilitating homologous recombination-dependent DNA damage repair. Cancer Lett. 2023;577:216440. 10.1016/j.canlet.2023.216440. 37838281 10.1016/j.canlet.2023.216440

