# Supplementary material for: USP10/XAB2/ANXA2 axis promotes DNA damage repair to enhance chemoresistance to oxaliplatin in colorectal cancer
Source: J Exp Clin Cancer Res. 2025 Mar 11;44:94. doi: 10.1186/s13046-025-03357-z (PMC11895293; doi:10.1186/s13046-025-03357-z)
Supplement: Supplementary file 1 — Supplementary Material 1: Supplementary Methods [file 13046_2025_3357_MOESM1_ESM.docx]

# Supplementary Methods

# Antibodies and reagents

The following antibodies were used: XAB2 (10637-1-AP), GAPDH (60004-1-Ig), γH2AX (ser139) (29380-1-AP), Ki-67 (28074-1-AP), PCNA (10205-2-AP), ANXA2 (60051-1-Ig), FLAG-tag (66008-4-Ig), MYC-tag (60003-2-Ig), Histone H3 (68345-1-Ig), Ubiquitin (10201-2-AP), and HA-tag (51064-2-AP) from Proteintech (Wuhan, China) and USP10 (ab70895) from Abcam (USA). Oxaliplatin (HY-17371), Spautin-1 (HY-12990), Cycloheximide (HY-12320), and Ku55933 (HY-12016) were purchased from MedChemExpress (USA). MG132 (S1748-25mg) was purchased from Beyotime Biotechnology (Shanghai, China).

# Plasmid construction, cell transfection, and lentivirus infection

XAB2-siRNA, ANXA2-siRNA, and USP10-siRNA (RiboBio, Guangzhou) and Flag-USP10-wild type (WT), Flag-USP10-mutant 1 (M1), Flag-USP10-mutant 2 (M2), Flag-USP10-mutant 3 (M3), Flag-USP10-C424A (a dominant negative form that lost its DUB activity), Myc-XAB2, HA-Ub, and XAB2 plasmids (Hanbio Biotechnology, Shanghai) were transfected using jetPRIME (Polyplus, France) according to the manufacturer's instructions. The transfection efficiency was measured using quantitative real-time PCR and western blot. ShXAB2, shANXA2, and shUSP10 lentiviruses (pHBLV-U6-MCS-PGK-Puromycin) were purchased from Hanbio Biotechnology (Shanghai, China), which were used to infect CRC cells separately according to manufacturer's instructions. Stable cell lines were selected by 3 ug/ml puromycin. The sequences of all siRNAs and shRNAs are showed as follows: Negative control (si-NC and sh-NC) sequence: 5′-TTCTCCGAACGTGTCACGTAA-3′; XAB2 (siRNA-1) sequences: 5′-GGAACCAATTCTCTGTCAA-3′; XAB2 (siRNA-2) sequences: 5′-AGGAGAGCTTCAAGGCGTA-3′; XAB2 (siRNA-3) sequences: 5′-ACGCAGCACTCTCGAATTT-3′; XAB2 (shRNA) sequences: 5′-AGGAGAGCTTCAAGGCGTA-3′; ANXA2 (siRNA-1) sequences: 5′-CGGCTGTATGACTCCATGA-3′; ANXA2 (siRNA-2) sequences: 5′-GACCAACCGCAGCAATGCA-3′; ANXA2 (siRNA-3) sequences: 5′-GTCTGTCAAAGCCTATACT-3′; ANXA2 (shRNA) sequences: 5′-GTCTGTCAAAGCCTATACT-3′; USP10 (siRNA-1) sequences: 5′-CCCTGATGGTATCACTAAA-3′; USP10 (siRNA-2) sequences: 5′-CCAAGGTTATACCACAAAA-3′; USP10 (siRNA-3) sequences: 5′-GCAGAATTTATGGGTGACA-3′; USP10 (shRNA) sequences: 5′-CCCTGATGGTATCACTAAA-3′.

# Quantitative real-time PCR (qRT-PCR) and RNA-sequencing (RNA-seq)

Total RNA was extracted from cells by Trizol reagent (Invitrogen). Extracted RNA was reversely transcribed to cDNA by using a PrimeScript RT Reagent Kit (Takara). The cDNA was quantified by qRT-PCR with ChamQ Universal SYBR qPCR Master Mix (Vazyme) on LightCycler® 96 Instrument (Roche), with GAPDH employed as an internal control. The primer sequences used for qRT-PCR are showed as follows: XAB2 (forward: 5′-AATCAGCTATACGAGCGGGC-3′; reverse: 5′-CTTCATAGGCAGGGTCGGTC-3′); MMP9 (forward: 5′-AGACCTGGGCAGATTCCAAAC-3′; reverse: 5′-CGGCAAGTCTTCCGAGTAGT-3′); ANXA2 (forward: 5′-TCTACTGTTCACGAAATCCTGTG-3′; reverse: 5′-AGTATAGGCTTTGACAGACCCAT-3′); DLX2 (forward: 5′-ATGCACTCGACCCAGATCG-3′; reverse: 5′-GGCTTGGTACTGGTAGGAACC-3′); GAPDH (forward: 5′-GCACCGTCAAGGCTGAGAAC-3′; reverse: 5′-TGGTGAAGACGCCAGTGGA-3′); ANXA2 promoter-P1 (forward: 5′-CTCACATTCCTCAGAAATAGCAAAC-3′; reverse: 5′-CACGATTCAGCCTCCCTT-3′); ANXA2 promoter-P2 (forward: 5′-CGGAAGAAAAGCGAGTAACA-3′; reverse: 5′-CGTAGCAGGCAGTCCTGAG-3′); ANXA2 promoter-P3 (forward: 5′-GTGGGCAGAGCACTGAATG-3′; reverse: 5′-CGCTGAGAATCCCGAGG-3′). RNA-seq was performed using total RNA extracts from SW480 cell lines transfected with shXAB2 and its control by Seqhealth Technology Co., LTD (Wuhan, China). After confirming the integrity and purity of RNA, we prepared an RNA-seq library using pre-processed RNA and performed data quality control using fastp (version 0.23.0). After filtering the original data, the clean data were adjusted to the reference genome with STAR (version 2.5.3a). Subsequently, statistically significant differentially expressed RNAs were determined by edgeR (version 3.12.1).

# Western blot

Mixed the IP lysis buffer (Beyotime, P0013), protease inhibitor cocktail (APExBIO, K1007), and phosphatase inhibitor cocktail (APExBIO, K1015) in a ratio of 100:1:1. Then lysed the cells on ice for 15 min, and centrifuged with 12,000 × g at 4 ° C for 15 min. The protein concentration was determined using the BCA protein assay kit (Beyotime, P0012). 30 μg protein was used as the sample to be separated on 7.5%-15% SDS-PAGE gel and transferred to PVDF membrane (Millipore, ISEQ00010). After block in 5% skimmed milk at room temperature for one hour, the membranes were incubated with different primary antibodies at 4 ° C overnight. After three washes, the membranes were incubated with a 1:10000 dilution of HRP-conjugated secondary antibodies for one hour at room temperature. Finally, using the enhanced chemiluminescence kit (Beyotime, P0018FS) to visualize the target protein.

# Immunohistochemistry (IHC) staining and evaluation

Pairs of paraffin-embedded CRC and adjacent non-neoplastic tissues were stained with 1:500 dilutions of anti‐XAB2 (Proteintech, 10637-1-AP) antibody. A DAB system (MXB Biotechnologies, KIT-9710) was used to identify the protein expression level. Sections were photographed under a microscope in five random areas, and then scored independently for staining intensity (0, negative staining; 1, weak staining; 2, moderate staining; 3, strong staining) and staining area (0, no staining; 1, 1%-25%; 2, 26%-50%; 3, 51%-75%; 4, 76%-100%) by two clinical pathologists. Finally, we evaluated the expression level of XAB2 based on the product of the staining intensity and the staining area. High expression was defined as a score of ≥5, and low expression was defined as a score of ≤4.

# Cell proliferation assays and in vitro half maximal inhibitory concentration (IC50) assays

For the cell proliferation assays, 3,000 cells were seeded into 96-well plates, cell viability was assessed for 5 consecutive days by the Cell Counting Kit-8 (APExBIO, K1018) according to the manufacturer's instructions. For cytotoxic assay, cells were cultured for 24 h and then various concentration of oxaliplatin was added for 48 h. The indicated OD value was measured at 450 nm, and the IC50 value was calculated using GraphPad Prism 9.0.

# Colony formation assays

2,000 cells were seeded in 6-well plates and were cultured for 2 weeks. Cells were washed with PBS, fixed with methanol and stained with crystal violet. Colonies with over 50 cells were counted as an individual using ImageJ.

# Apoptosis assays

Cells were seeded in 12-well plates, treated with or without oxaliplatin (7.5μM) for 48h. Cells were harvested and resuspended in 500 μl binding buffer containing 5 μl Annexin V-FITC and 5 μl propidium Iodide (PI) (APExBIO, K2003) at room temperature in the dark for 20 min. The samples were analyzed by FACSymphony A1 flow cytometer (Becton Dickson). The total apoptotic rate includes the sum of early and late apoptotic cells.

# Immunofluorescent

Cells were seeded in 12-well plates for 24h and treated with or without oxaliplatin (7.5μM) for 24h, or treated with 20mM Ku55933 (ATM specific inhibitor) for 1h then with oxaliplatin (7.5μM) for 24h. Then cells were fixed with 4% paraformaldehyde for 15 min and permeabilized with 0.5% Triton X-100 for 10 min at room temperature. After that, cells were blocked with immunol staining blocking buffer (Beyotime, P0102) at room temperature for 1 hour, and incubated overnight at 4 ° C with primary antibodies against γH2AX or USP10 and incubated for 1 hour at room temperature with secondary antibody Alexa Fluor 488-conjugated anti-rabbit IgG (Beyotime, A0423). Nucleus were counterstained with DAPI (Beyotime, C1005). Finally, images were observed by an Olympus IX74 fluorescence microscope.

# Alkaline comet assays

Single cell DNA damage detected by alkaline comet assay. Cells were trypsinized and resuspended to a concentration of 1×10^6^/ml. We used the DNA damage comet assay kit (Beyotime, C2041M), according to the manufacturer’s instructions, we first prepared 1% normal melting point agarose gel, and then mixed 10,000 cells with 0.7% low melting point agarose gel. Cells were lysed, then subjected to electrophoresis under alkaline conditions and stained with PI. Finally, images were captured using Olympus IX74 fluorescence microscope, and tail moment and tail DNA (%) were calculated using CASP software to measure the degree of DNA damage.

# Chromatin immunoprecipitation-sequencing (ChIP-seq) and ChIP‒qPCR

The ChIP-seq was conducted by Seqhealth Technology Co., LTD (Wuhan, China). After the steps of cell cross-linking, cell lysis, protein immunoprecipitation, and DNA extraction, the high-throughput DNA sequencing libraries were prepared by using VAHTS Universal DNA Library Prep Kit for Illumina V3 (Vazyme, ND607). The library products corresponding to 200-500 bps were enriched, quantified and finally sequenced on DNBSEQ-T7 sequencer (MGI Tech Co., Ltd. China) with PE150 model. ChIP assays were performed using a ChIP Assay kit (Beyotime, P2078) according to the manufacturer’s instructions. In short, cells were cross-linked in 1% formaldehyde at 37°C for 10 min, lysed using SDS Lysis Buffer containing 1mM PMSF, and sonicated to cleave genomic DNA. Then mixed the sample with NaCl and heated it at 65 ° C for 4 h to remove cross-linking. Finally, used the specific primers shown in Table. S3 to analyze the eluted DNA fragments by qPCR.

# Dual-luciferase reporter assays

The ANXA2 wild type promoter and ANXA2 mutant (MUT) promoter were cloned into the pGL3-Basic vector (GenePharma, China). HEK-293T cells were seeded in a 24-well plate for 24 hours. The indicated plasmids were cotransfected with pRL-TK Renilla plasmid using jetPRIME (Polyplus, France). The dual-luciferase reporter assay was conducted using a Dual Luciferase Reporter Assay Kit (Vazyme, DL101-01). Results were normalized for expression of pRL-TK as measured by Renilla luciferase activity.

# Co-immunoprecipitation (Co-IP) and mass spectrometry (MS)

For endogenous Co-IP assay, cells were lysed using IP lysis buffer, 5ug of antibody was added to the cells, and incubated overnight at 4 °C. Then, added 0.2mg of protein A+G beads (Beyotime, P2108) and incubated at room temperature for 1 hour. For exogenous Co-IP assay, experiments were conducted using anti-Flag beads (Beyotime, P2115) and anti-Myc beads (Beyotime, P2118). Then used antibodies against Flag and Myc to immunoprecipitate proteins and eluted protein complexes. The eluate was collected and visualized on the SDS gel, and then silver staining was performed with a fast silver stain kit (Beyotime, P0017S). Retrieve and analyze different protein bands using MS.
