# Supplementary material for: USP10/XAB2/ANXA2 axis promotes DNA damage repair to enhance chemoresistance to oxaliplatin in colorectal cancer
Source: J Exp Clin Cancer Res. 2025 Mar 11;44:94. doi: 10.1186/s13046-025-03357-z (PMC11895293; doi:10.1186/s13046-025-03357-z)
Supplement: Supplementary file 2 — Supplementary Material 2: Table S1: Association between XAB2 expression and clinicopathological parameters in colorectal cancer patients. [file 13046_2025_3357_MOESM2_ESM.docx]

**Table S1. Association between XAB2 expression and clinicopathological parameters in colorectal cancer patients.**

| **Parameters** | **Cases** | **XAB2 expression** | | **χ2** | ***P*** |
| --- | --- | --- | --- | --- | --- |
|  |  | **High** | **Low** |  |  |
| **Age（years）** |  |  |  |  |  |
| ≤65 | 20 | 11 | 9 | 0.333 | 0.564 |
| >65 | 30 | 14 | 16 |  |  |
| **Gender** |  |  |  |  |  |
| Female | 24 | 11 | 13 | 0.703 | 0.402 |
| Male | 26 | 15 | 11 |  |  |
| **Tumor invasion depth** |  |  |  |  |  |
| T1+T2 | 16 | 6 | 10 | 5.995 | 0.014 |
| T3+T4 | 34 | 25 | 9 |  |  |
| **Lymph node metastasis** |  |  |  |  |  |
| N0 | 28 | 15 | 13 | 0.152 | 0.696 |
| N1+N2 | 22 | 13 | 9 |  |  |
| **Distant metastasis** |  |  |  |  |  |
| M0 | 44 | 25 | 19 | 0.100 | 0.752 |
| M1 | 6 | 3 | 3 |  |  |
| **TNM** |  |  |  |  |  |
| I+II | 27 | 10 | 17 | 5.265 | 0.022 |
| III+IV | 23 | 16 | 7 |  |  |
