# Supplementary figures and images for: USP10/XAB2/ANXA2 axis promotes DNA damage repair to enhance chemoresistance to oxaliplatin in colorectal cancer
Source: J Exp Clin Cancer Res. 2025 Mar 11;44:94. doi: 10.1186/s13046-025-03357-z (PMC11895293; doi:10.1186/s13046-025-03357-z)

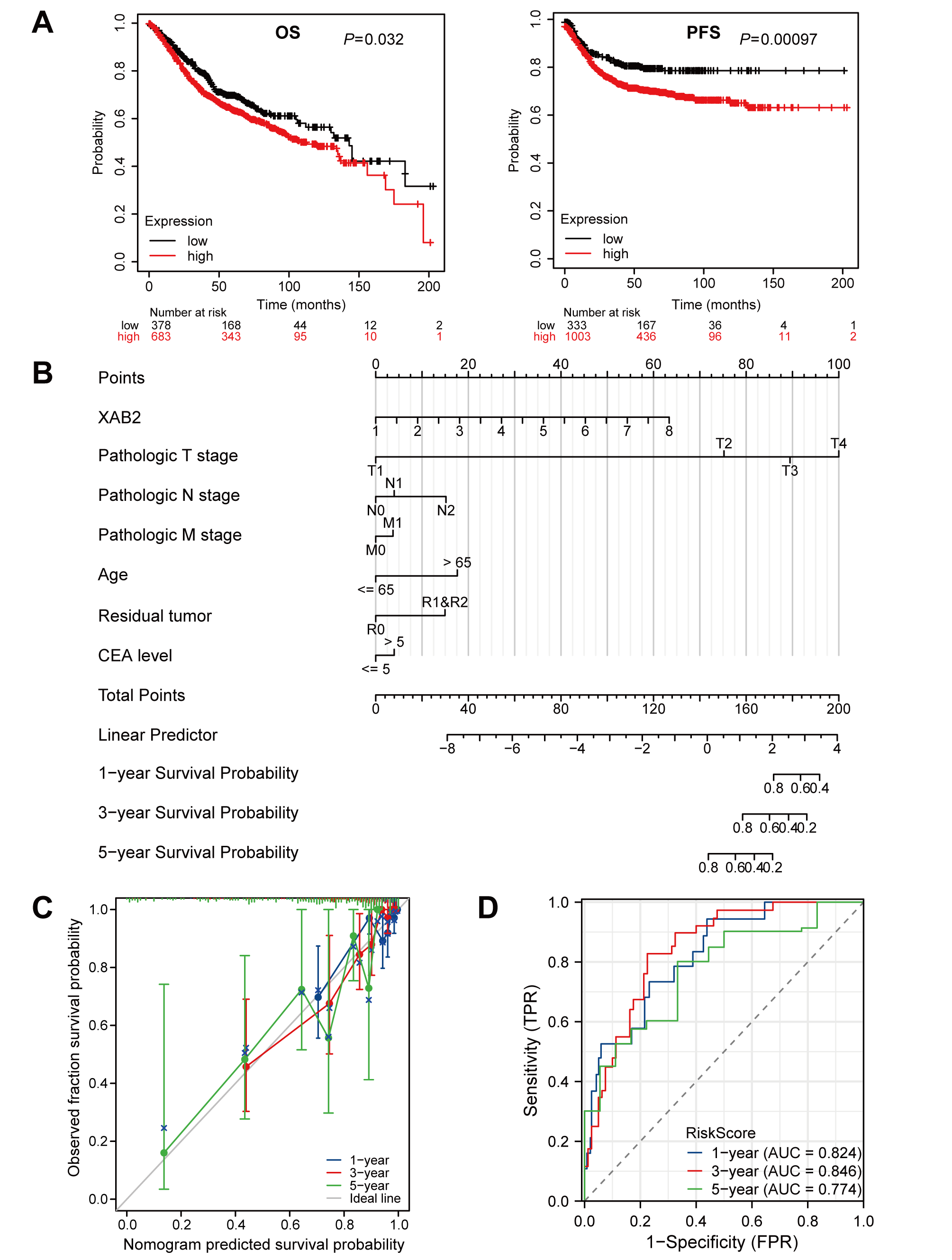

Supplement: Supplementary file 3 — Supplementary Material 3: Figure S1: Construction and validation of prognostic model based on XAB2. A Kaplan–Meier analysis showing OS and PFS curves of patients with CRC stratified by high versus low XAB2 expression from Kaplan-Meier plotter website. B Nomogram Predicting OS for patients with CRC in TCGA Cohorts. C The calibration curve of the nomogram. D The time-dependent ROC curve of the nomogram. [file 13046_2025_3357_MOESM3_ESM.tif]

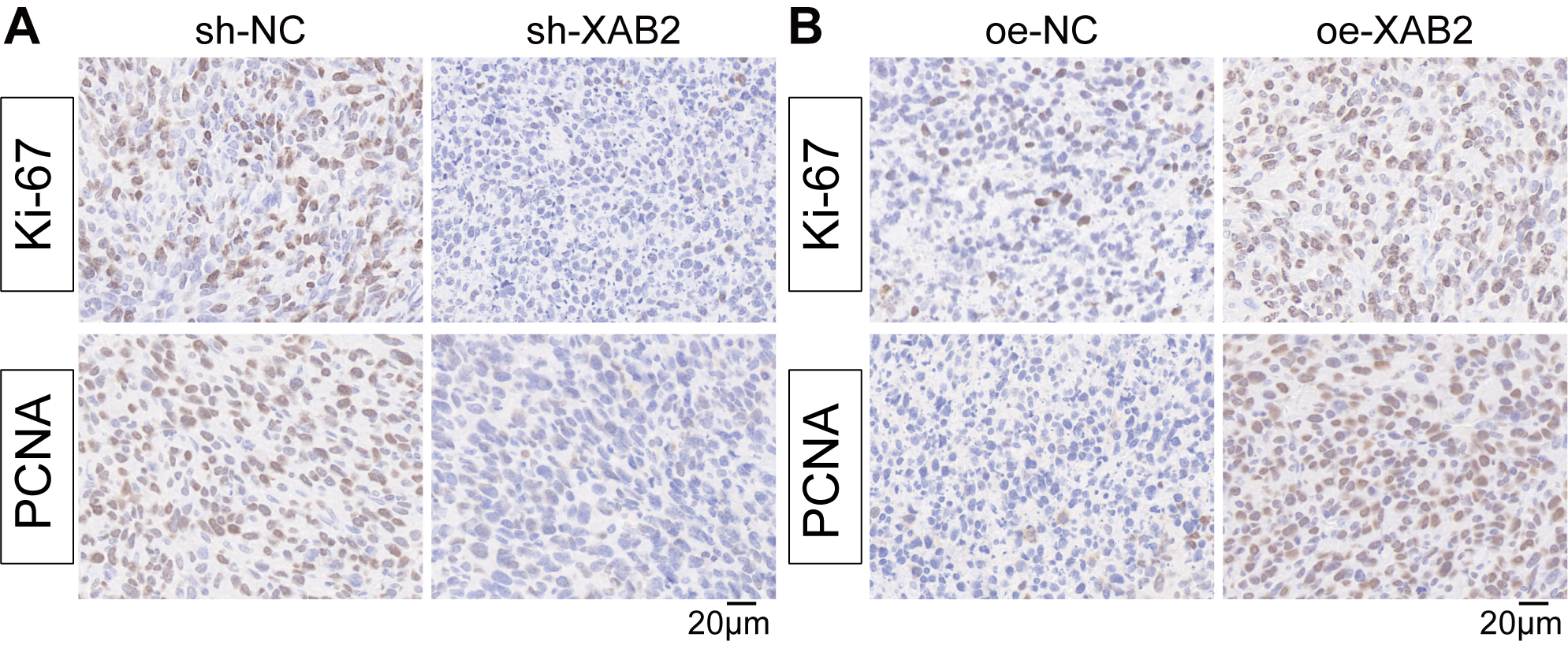

Supplement: Supplementary file 4 — Supplementary Material 4: Figure S2: XAB2 enhances the expression of ki-67 and PCNA in vivo. A-B Representative IHC images of ki-67 and PCNA expression in xenograft tumor tissues from the indicated groups. [file 13046_2025_3357_MOESM4_ESM.tif]

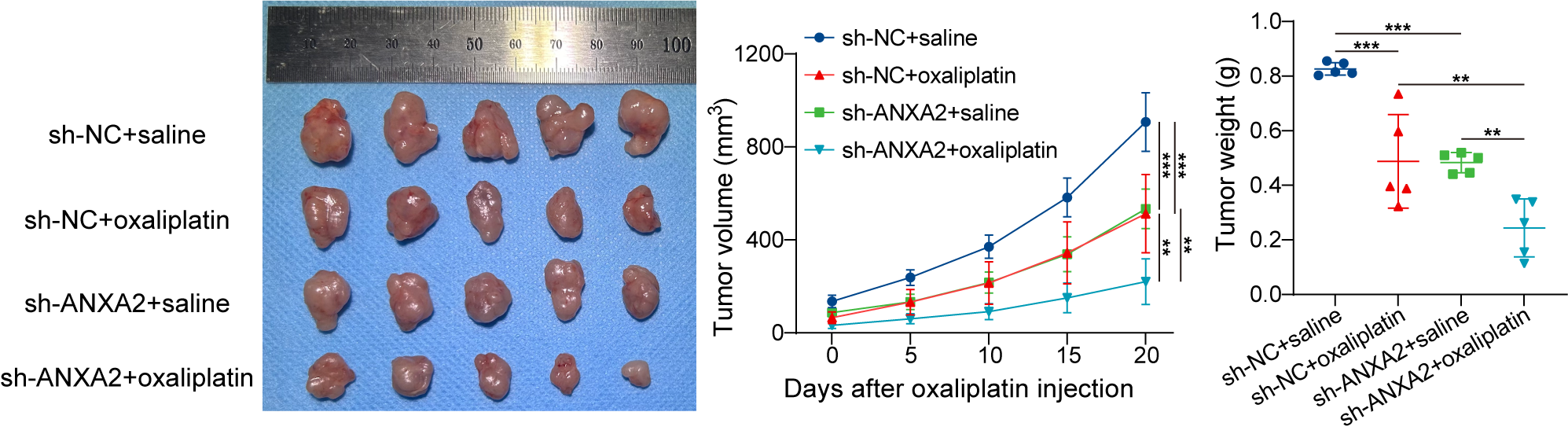

Supplement: Supplementary file 5 — Supplementary Material 5: Figure S3: ANXA2 increases the oxaliplatin resistance of CRC cells in vivo. Transplanted xenografts derived from cells with sh-NC and sh-ANXA2 were established in BALB/c nude mice (n = 5). One week after injection, oxaliplatin (20 mg/kg) or an equivalent volume of saline was injected into the tumor every 5 days for 20 days. Tumor volume and weight were measured. Data are presented as mean ± SD (**P < 0.01, ***P < 0.001). [file 13046_2025_3357_MOESM5_ESM.tif]
